# Thermoresponsive Brush Coatings for Cell Sheet Engineering with Low Protein Adsorption above the Polymers’ Phase Transition Temperature

**DOI:** 10.1021/acsabm.4c01127

**Published:** 2024-11-05

**Authors:** Alexander Schweigerdt, Daniel D. Stöbener, Johanna Scholz, Andreas Schäfer, Marie Weinhart

**Affiliations:** † Institute of Chemistry and Biochemistry, Freie Universitaet Berlin, Takustr. 3, 14195 Berlin, Germany; ‡ Institute of Physical Chemistry and Electrochemistry, Leibniz Universitaet Hannover, Callinstr. 3A, 30167 Hannover, Germany

**Keywords:** functional coatings, LCST-type polymer, antifouling, cell sheet fabrication, sterilization

## Abstract

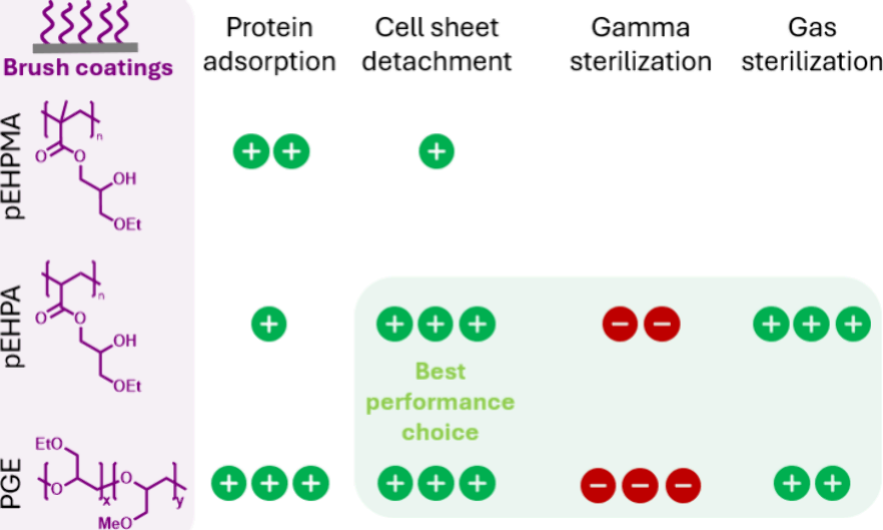

Thermoresponsive polymer coatings on cell culture substrates enable noninvasive cell detachment and cell sheet fabrication for biomedical applications. Optimized coatings should support controlled culture and detachment of various cell types and allow chemical modifications, e.g., to introduce specific growth factors for enhanced gene expression. Furthermore, the sterilization and storage stability of the coatings must be assessed for translational attempts. Poly(glycidyl ether) (**PGE**) brush coatings with short alkoxy side chains provide a versatile platform for cell culture and detachment, but their polyether backbones are susceptible to oxidation and degradation. Thus, we rationally designed potential alternatives with thermoresponsive glycerol-based block copolymers comprising a stable polyacrylate or polymethacrylate backbone and an oligomeric benzophenone (BP)-based anchor. The resulting poly(ethoxy hydroxypropyl acrylate-*b*-benzophenone acrylate) (**pEHPA-***b***-BP**) and poly(ethoxy hydroxypropyl methacrylate-*b*-benzophenone methacrylate) (**pEHPMA-***b***-BP**) block copolymers preserve the short alkoxy-terminated side chains of the **PGE** derived structure on a stable, but hydrophobic, aliphatic backbone. The amphiphilicity balance is maintained through incorporated hydroxyl groups, which simultaneously can be used for chemical modification. The polymers were tailored into brush coatings on polystyrene surfaces via directed adsorption using the BP oligomer anchor. The resulting coatings with thickness values up to ∼3 nm supported efficient adhesion and proliferation of human fibroblasts despite minimal protein adsorption. The conditions for cell sheet fabrication on **pEHPA-***b***-BP** were gentler and more reliable than on **pEHPMA-***b***-BP**, which required additional cooling. Hence, the stability of **pEHPA-***b***-BP** and **PGE** coatings was evaluated post gamma and formaldehyde (FO) gas sterilization. Gamma sterilization partially degraded **PGE** coatings and hindered cell detachment on **pEHPA-***b***-BP**. In contrast, FO sterilization only slowed detachment on **PGE** coatings and had no adverse effects on **pEHPA-***b***-BP**, maintaining their efficient performance in cell sheet fabrication.

## Introduction

Polymer coatings with a lower critical solution temperature (LCST) behavior enable gentle, enzyme-free cell harvesting *in vitro*. These coatings promote cell adhesion and proliferation above the polymers’ LCST at physiological conditions (37 °C) and become cell-repellent below their LCST, allowing controlled cell detachment without damaging cells or the extracellular matrix (ECM).^1,2^

The properties of LCST-type polymers can be tailored through monomer selection to direct the location of their phase transitions in aqueous solution to the physiological temperature range^3−5^ and fine-tune their amphiphilic balance through copolymerization.^6,7^ Functional polymer coatings, exhibiting a phase transition on the surface of cell culture materials, can be designed with brush, bottlebrush, or hydrogel architecture.^8−11^ At physiological conditions, these coatings should be in a dehydrated, collapsed state to promote serum protein adsorption, which mediates cell adhesion of anchorage-dependent cells.^12,13^ Nonspecific serum protein adsorption on artificial surfaces occurs spontaneously and requires minimal exposure to enable cell-surface interactions.^13,14^ For instance, *Horbett et al.* demonstrated that fibroblasts spread similarly on substrates exposed to serum for either 10 s or 90 min.^15^ Adsorbed fibronectin (Fn) and vitronectin from serum-containing medium mediate cell adhesion via integrin-binding RGD sequences, provided they remain structurally intact.^16^ Upon RGD-binding of anchorage-dependent cells, adhesion and proliferation processes, such as cytoskeleton reorganization and production of ECM proteins, are initiated.^17,18^

For efficient thermally induced cell detachment, the thermoresponsive coating or at least its outermost layer^19^ must transition into a rehydrated state upon cooling. Concomitantly, interactions between the cell-derived ECM and the culture substrate become unfavorable, leading to ECM-substrate separation.^13,19^ For practical convenience, detachment at room temperature (20–25 °C) is preferred, although successful detachment procedures at temperatures below 20 °C were also developed.^20,21^ Thermoresponsive polymer coatings generally exhibit broader phase transitions compared to their aqueous polymer solutions due to restricted chain movement after surface tethering.^19,22,23^ Additionally, phase transition temperatures can be influenced through the coating-substrate or polymer-salt and polymer–protein interactions.^11,24,25^ Consequently, inhomogeneous layer (de)hydration and hampered phase transitions must be considered when optimizing the coating performance for cell sheet fabrication. For example, poly(*N*-isopropylacrylamide) (**pNIPAM**, LCST ∼ 32 °C) hydrogel and poly(oligo ethylene glycol methacrylate) (**pOEGMA**) (LCST ∼ 28–34 °C) brush coatings can lack cell adhesive properties above their phase transition temperature^9,26−28^ and poly(glycidyl ether) (**PGE**) brushes (LCST ∼ 20–30 °C) may not reliably mediate controlled cell sheet detachment at distinct grafting densities.^11^

The performance of thermoresponsive coatings can be further optimized by incorporating functional units. For instance, copolymers of 2-carboxy isopropylacrylamide with **NIPAM** were functionalized with heparin using the carboxyl group, which enabled the binding of epidermal growth factors for enhanced expression of hepatocyte-specific genes in primary hepatocytes.^29^ Therefore, polymers with accessible reactive groups, e.g., hydroxyl, carboxyl, or amine groups, are desirable.^30,31^ However, most established thermoresponsive polymers, such as **pNIPAM**, **pOEGMA**, or poly(oxazoline)s (**POX**) lack reactive groups and require copolymerization, which may impact their LCST behavior.^32^ Additionally, stability during sterilization is crucial for technology translation into biomedical laboratories and potential product development. While **pNIPAM** surfaces are compatible with gas sterilization, frequently used ionizing radiation increases the LCST or induces cross-linking in **pNIPAM** or **POX** polymers, thus potentially limiting their phase transition and application in the physiological range.^33,34^

**Figure 1:**
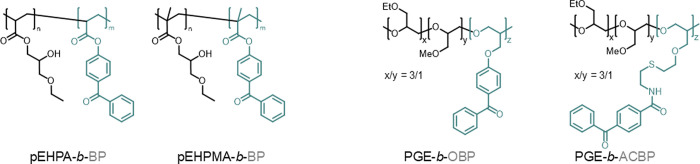
Chemical structures of the glycerol-based thermoresponsive copolymers **pEHPA-***b***-BP** and **pEHPMA-***b***-BP**, which were used to functionally coat PS surfaces in this work (left), as well as **PGE-***b***-OBP** and **PGE-***b***-ACBP**, which were used for comparison as established (TC)PS coating systems (right).

In previous work, we established thermoresponsive poly(glycidyl ether) (**PGE**) coatings optimized for cell sheet fabrication.^35^ Their alkoxy side groups induce LCST behavior, allowing thermal control over cell adhesion, though lacking postfunctionalization options.^35,36^ Limitations in the shelf life and sterilization stability of structurally similar poly(ethylene glycol) (**PEG**) coatings further raise concerns about the stability of thermoresponsive **PGE** brushes under storage or radiation sterilization conditions.^37−39^ Therefore, we applied molecular design principles to conceptualize, synthesize, and characterize poly(γ-alkoxy-β-hydroxy-(meth)acrylate) polymers, incorporating stable aliphatic backbones and hydroxyl groups for postfunctionalization while preserving **PGE**-like short alkoxy side chains.^3^ The resulting poly(hydroxy methoxypropyl methacrylate) (**pHMPMA**), however, exhibited cloud point temperatures (*T*_cp_) mainly above the physiological range (37–51 °C). Furthermore, no distinct microscopic dehydration was detected, which is an important prerequisite for cell culture applications.^3^ In contrast, poly(ethoxy hydroxypropyl acrylate) (**pEHPA**) homopolymers exhibited *T*_cp_ values of 22–27 °C and distinct chain dehydration above the *T*_cp_, thus showing potential for a functional cell sheet fabrication coating.^3^ To develop functional brush coatings via block copolymer self-assembly on PS substrates in this work, we prepared thermoresponsive block copolymers with a poly(meth)acrylate backbone comprising ethoxy hydroxypropyl side chains, avoiding methoxy side chains, which have been shown to introduce unfavorable thermoresponsive properties^3^ to the polymer (Figure 1).

Both thermoresponsive poly(ethoxy hydroxypropyl acrylate-*b*-benzophenone acrylate) (**pEHPA-***b***-BP**) and poly(ethoxyhydroxypropyl methacrylate-*b*-benzophenone methacrylate) (**pEHPMA-***b***-BP**) were designed with a short benzophenone (BP)-comprising block for efficient self-assembly and covalent surface attachment. The performance of the resulting brush coatings was evaluated in cell culture. Cell sheet fabrication on these surfaces was investigated before and after formaldehyde (FO) gas and gamma sterilization and compared to established **PGE-***b***-OBP** and **PGE-***b***-ACBP** brush coatings on PS and TCPS (Figure 1).^11^**pEHP[M]A**-based functional coatings showed efficient cell adhesion despite striking differences in protein adsorption, compared to **PGE**, and the aliphatic backbone further enhanced poststerilization performance.

## Experimental Section

Materials, synthetic pathways and procedures, as well as the characterization of the used monomers (Scheme S1) and polymers (Scheme S2), including gel permeation chromatography (GPC) methods, sample preparation for thermoresponsive analysis, additional UV–vis, dynamic light scattering (DLS), and nuclear magnetic resonance (NMR) spectroscopy data are presented in the electronic Supporting Information (SI). Procedures for the preparation of PS-coated surfaces on silicon wafers or quartz crystal microbalance (QCM-D) chips are also provided in the SI.

### Turbidimetry (UV–vis) Measurements

Absorbance and transmittance measurements were recorded on a PerkinElmer Lambda 950 UV–vis spectrometer with a PTP 6 Peltier temperature programmer (PerkinElmer). Temperature-dependent measurements were performed at heating rates of 0.5 °C min^–1^ while recording data points every 0.5 °C. The temperature-dependent transmittance of the aqueous polymer solution was measured for two cycles per sample. The measurement points were connected via the Akima-spline interpolation, and the cloud point temperature *T*_cp_ was defined as the temperature at the inflection point of the normalized transmittance versus temperature curve.

### Temperature-Dependent NMR Measurements

^1^H and ^13^C NMR spectra were recorded on a JEOL ECZ operating at 600 and 150 MHz, respectively. Deuterated water (D_2_O) was filtered over a 0.45 μm syringe filter (cellulose acetate), and samples were prepared at a concentration of 10 mg mL^–1^.

### Preparation of Polymer Solutions and Fabrication of Brush Coatings

Milli-Q water and absolute ethanol were used for all polymer solutions. The preparation of thermoresponsive **PGE** brush coatings was described in detail previously.^11,40^ Briefly, (TC)PS model substrates, placed in PS Petri dishes, or plain (TC)PS Petri dish surfaces were incubated in **PGE**-block copolymer solution (2 mL, 250 μg mL^–1^) for 60 min. A **PGE-***b***-OBP** solution in 54/46 water/ethanol (v:v) or a **PGE-***b***-ACBP** solution in 52/48 water/ethanol (v:v) was used for the self-assembly process on PS and TCPS substrates, respectively. After incubation, the polymer solution was discarded, the coatings briefly washed with water, dried under a nitrogen stream, and irradiated under UV light (UV KUB, LED, λ = 365 nm) for 160 s to covalently attach the adsorbed polymer brushes to the substrate.

**pEHP[M]A-***b***-BP** polymer solutions in 55/45 water/ethanol (v:v) were prepared at a concentration of 250 or 62.5 μg mL^–1^. Before the adsorption step, the polymer solutions (2.5 mL per substrate) gauged in a syringe were equilibrated at 35 °C for 20 min together with the dry model PS substrates or PS Petri dishes. Afterward, the polymer solutions were filtered (0.22 μm cellulose acetate syringe filter) onto substrates, and the incubation procedure proceeded for 60 min at 35 °C. After incubation, the polymer solutions were discarded, and the brush coatings were briefly washed with water at room temperature (RT), dried under a nitrogen stream, and irradiated under UV light (UV KUB, LED, λ = 365 nm) for 16 min to covalently attach the adsorbed brushes to the substrate.

After irradiation, all brush-coated surfaces were immersed in ethanol for at least 16 h to remove noncovalently attached chains from the surface and dried under a stream of nitrogen before characterization and usage in further experiments.

### Brush Coating Analysis

Coating thickness in the dry state was analyzed through spectroscopic ellipsometry measurements and evaluated via a Cauchy fit. A fixed refractive index *R*_f_ = 1.45 was used for **PGE** brush coatings,^40^ while for **pEHP[M]A** brush coatings, an *R*_f_ = 1.51 was approximated due to the structural resemblance to poly(hydroxyethyl methacrylate) (**pHEMA**).^41,42^ Water contact angles (CA) were measured at ambient conditions (20 °C) via the sessile drop method (2 μL) applying the Laplace–Young model.

### Quartz Crystal Microbalance with Dissipation (QCM-D) Measurements for Quantification of Protein Adsorption

Protein adsorption from standard Dulbecco’s modified eagle medium (DMEM) supplemented with 10% fetal bovine serum (FBS) (v:v) on **pEHP[M]A** brushes and TCPS controls was measured at 20 and 37 °C under a constant flow of 0.1 mL min^–1^. After thermal surface equilibration under phosphate buffered saline (PBS) flow, the medium was changed to serum protein-containing DMEM medium for 20 min and flushed again with PBS until the signal remained constant (5 min). The frequency differences Δ*f* of the third overtone before and after protein exposure to the surface were converted to areal mass coverage using the Sauerbrey equation and the fundamental QCM-D chip frequency of 4.95 MHz to access the adsorbed areal protein mass. Representative frequency curves are shown in Figure S12.

### Cell Culture Conditions

Detailed cell culture conditions are described in the SI. For adhesion studies on **pEHP[M]A** coatings, human dermal fibroblasts (HDF) were seeded with a density of 43 × 10^5^ cells cm^–2^, and phase contrast images were taken after 1, 4, 24, 48, and 72 h. For cell detachment studies on **PGE** coatings, HDF were seeded with a density of 160 × 10^5^ cells cm^–2^, and phase images were taken after 24 h, as established previously.^19^ For cell detachment studies on **pEHP[M]A** coatings, HDFs were seeded with a density of 104 × 10^5^ cells cm^–2^ and phase images were taken after 24 and 48 h for **pEHPA-***b***-BP** and additionally after 72 h for **pEHPMA-***b***-BP**.

### Cell Detachment

Thermal detachment of cell sheets on **pEHPA-***b***-BP**, **PGE-***b***-OBP**, and **PGE-***b***-ACBP** coatings was triggered as follows: after reaching confluency, the cell culture medium was exchanged against PBS at RT for 10 min. Afterward, the PBS was exchanged for warm PBS (37 °C), and the dishes were placed in the cell culture incubator for 10 min. Finally, cell sheet detachment was observed under ambient conditions.

Thermal detachment on **pEHPMA-***b***-BP** coatings was triggered as follows: after reaching confluency, the cell culture medium was exchanged against 4 °C PBS with incubation for 10 min at 4 °C. Then, the PBS was exchanged for warm PBS (37 °C), and the dishes were placed in the cell culture incubator for 10 min. Finally, cell sheet detachment was observed under ambient conditions.

### Sterilization of Substrates

Surface disinfection or sterilization of **PGE** and **pEHP[M]A** brush coatings were performed via aq. EtOH (70 v-%) treatment, FO gas sterilization, and gamma radiation. Disinfection was performed by fully immersing the coated dishes in aq. EtOH for 10 min, followed by a 2-fold PBS wash afterward. Gas sterilization was performed with a Euro Formomat 5 device from MMM Group (München, Germany) using water-based 3% formaldehyde gas at 200 mbar and 60 °C for 6 h, according to the DIN EN 14180. Gamma sterilization was performed by BBF Sterilisationsservice GmbH (Kernen, Germany). Cobalt-60 was used as a source for gamma radiation, and a total dose of 45 kGy ± 10% was applied to the substrates, according to the DIN EN ISO 11137–1.

### Data Evaluation

Raw data processing and evaluation were performed with OriginPro and Microsoft Excel. Statistical comparison was performed using the Mann–Whitney-U test for two independent sample sets (ns, *p* > 0.1; *, *p* < 0.1; **, *p* < 0.05).

## Results and Discussion

### Polymer Synthesis and Characterization

The synthesis of **pEHPA** and **pEHPMA** homopolymers was performed according to previously established conditions for the controlled reversible addition–fragmentation transfer (RAFT) polymerization of **pEHPA**.^3^ Due to the poor solubility of the corresponding **pEHPMA** polymer in water, the polymerization of EHPMA was performed in dimethylformamide instead of water/1,4-dioxane mixture (Scheme S2a). The RAFT polymerization system allowed a convenient chain extension using benzophenone (BP) acrylate or methacrylate as comonomers (Scheme S1b,c and S2b,c) with overall isolated copolymer yields above 80%. The block copolymers **pEHPA-***b***-BP** and **pEHPMA-***b***-BP** comprise a short functional surface anchoring block with 2–4 BP units. Efficient attachment of the BP monomers to the homopolymer chains was indicated through the systematic increase of the whole distribution curve in the UV detector signal of the GPC elugrams (Figure S1). The structural characteristics of the synthesized polymers are listed in Table 1.

**Table 1:** Number Average Molecular Weight *M*_n_, Dispersity *Đ,* Number of BP Units Per Anchor Block, and Isolated Yield of the Synthesized Polymers after Dialysis

Polymer	*M*_n_ [kDa]^a^	*Đ*^a^	BP units^b^	Yield [%]^c^
**pEHPA**	22.6	1.15		69
**pEHPMA**	21.7	1.16		89
**pEHPA-***b***-BP**	24.1	1.25	3.9	87
**pEHPMA-***b***-BP**	22.1	1.18	2.4	88

a Derived from GPC measurements in THF calibrated with PMMA standards.

b Calculated from ^1^H NMR measurements.

c Monomer to polymer yield ratio.

The narrow dispersities ≤1.25 of the homo- and copolymers with molecular weights around 23 kDa indicate controlled polymerization conditions, essential for block copolymer synthesis close to the targeted value of 30 kDa. The BP-based anchor block enables directed physical adsorption of the block copolymers from selective solvents to hydrophobic surfaces such as PS via hydrophobic and π–π interactions. Furthermore, BP units promote covalent attachment of the assembled polymer chains to the PS surface via C–H insertion upon UV exposure,^40,43^ offering a convenient material-efficient and geometry-independent surface modification strategy.

### Evaluation of Thermoresponsive Behavior

**Figure 2:**
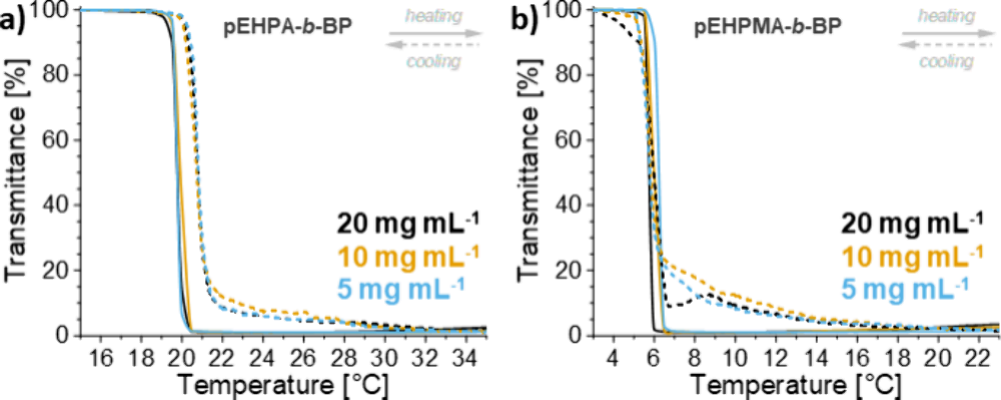
Representative concentration-dependent turbidimetry curves of **pEHPA-***b***-BP** (a) and **pEHPMA-***b***-BP** (b) in PBS after normalization from heating and cooling cycles (*n* = 2 per cycle).

The transition range of aqueous **pEHPA** solutions with a concentration between 20 and 5 mg mL^–1^ is located between 22 and 27 °C, comprising sharp phase transitions and a distinct dehydration behavior of the hydroxy groups as determined previously.^3^ Concentration-dependent turbidity measurements of analogous aqueous **pEHPMA** solutions (Figure S2) also indicate a sharp and reversible phase transition behavior. However, the more hydrophobic methacrylate backbone reduced the phase transition regime of the aqueous **pEHPMA** solutions to 8–11 °C with only a minor impact of salts on the *T*_cp_ (Table 2). Additional DLS measurements revealed solvated **pEHPMA** chains of ∼7 nm size at 8 °C, which increased to around 4800 nm in PBS and 630 nm in water upon thermally induced aggregation at RT (Figure S4). To further ensure that the hydrophobic BP block in **pEHPA-***b***-BP** and **pEHPMA-***b***-BP** is not adversely impacting the copolymers’ transition behavior, concentration-dependent turbidity studies were performed in PBS (Figure 2).

**Table 2:** Concentration-Dependent Cloud Point Temperatures *T*_cp_ Stated as Average Values ± Standard Deviation (SD) from Heating and Cooling Cycles of Thermoresponsive Homo- and Copolymers in PBS and Water (*n* = 4)^a^

Polymer	*T*_cp_ (20 mg mL^–1^) [°C]	*T*_cp_ (10 mg mL^–1^) [°C]	*T*_cp_ (5 mg mL^–1^) [°C]
**pEHPA** (H_2_O)^b^	23.2 ± 0.5	23.7 ± 0.5	24.5 ± 0.5
**pEHPA** (PBS)^b^	21.5 ± 0.5	22.0 ± 0.5	22.5 ± 0.5
**pEHPA-***b***-BP** (H_2_O)	22.3 ± 0.5	22.5 ± 0.5	23.3 ± 0.5
**pEHPA-***b***-BP** (PBS)	20.3 ± 0.6	20.3 ± 0.5	20.3 ± 0.6
**pEHPMA** (H_2_O)	9.0 ± 0.5	9.8 ± 0.5	10.3 ± 0.5
**pEHPMA** (PBS)	8.4 ± 0.6	8.8 ± 0.6	8.8 ± 0.5
**pEHPMA-***b***-BP** (H_2_O)^c^	7 ± 0.5	7.5 ± 0.5	7.6 ± 0.5
**pEHPMA-***b***-BP** (H_2_O)^d^	10.5 ± 0.8	13.9 ± 1.9	14.3 ± 1.3
**pEHPMA-***b***-BP** (PBS)	5.8 ± 0.5	6 ± 0.5	5.9 ± 0.5

a The minimal SD was aligned with the experimental error (±0.5 °C).

b Literature data obtained from Schweigerdt et al.^3^

c Value refers to the first of the two observed *T*_cp_’s

d Value refers to the second of the two observed *T*_cp_’s.

Both block copolymers exhibit concentration-independent, sharp, and reversible transitions in PBS over the scanned temperature and concentration range. A minor increase in transmittance during cooling cycles can be attributed to irreversible polymer precipitation due to a salting-out effect, which slightly decreases the polymer concnetration.^3,24^ While the presence of salts hardly altered the phase transition behavior of **pEHPA-***b***-BP** solutions, a significant impact was observed for **pEHPMA-***b***-BP** when comparing turbidity curves in water (Figure S3) and PBS (Figure 2). In water, two sigmoidal transition regions were detected between 7 and 8 °C and 10–14 °C, corresponding to a polymer aggregate size of 37 and 67 nm (Figure S5), respectively. In the presence of ions, the two transition regimes unified to a single cloud point at approximately 6 °C. The first cloud point in water can be attributed to the phase transition of the **pEHPMA** block, since the *T*_cp_ values are located between the ones of **pEHPMA** in H_2_O (9–10 °C) and **pEHPMA-***b***-BP** in PBS (∼6 °C). The hydrophobic **BP-**based block then stabilizes the aggregates for temperatures up to ∼14 °C in agreement with DLS data (Figure S5), before the transition continues, resulting in larger aggregates. An overview of the average *T*_cp_ values of the aqueous polymer solutions, including *T*_cp_ values of pEHPA solutions,^3^ is presented in Table 2.

The *T*_cp_ values of the block copolymers decrease by ∼2–3 °C compared to respective homopolymers due to the hydrophobic benzophenone block.^3^ Only a minimal concentration dependence of the *T*_cp_ values was observed, spanning ∼1 °C between 5 and 20 mg mL^–1^, except for the bimodal transition of **pEHPMA-***b***-BP** in water. As expected, the presence of ions in PBS and the hydrophobic BP block caused a minor *T*_cp_ decrease.^3,40^

**Figure 3:**
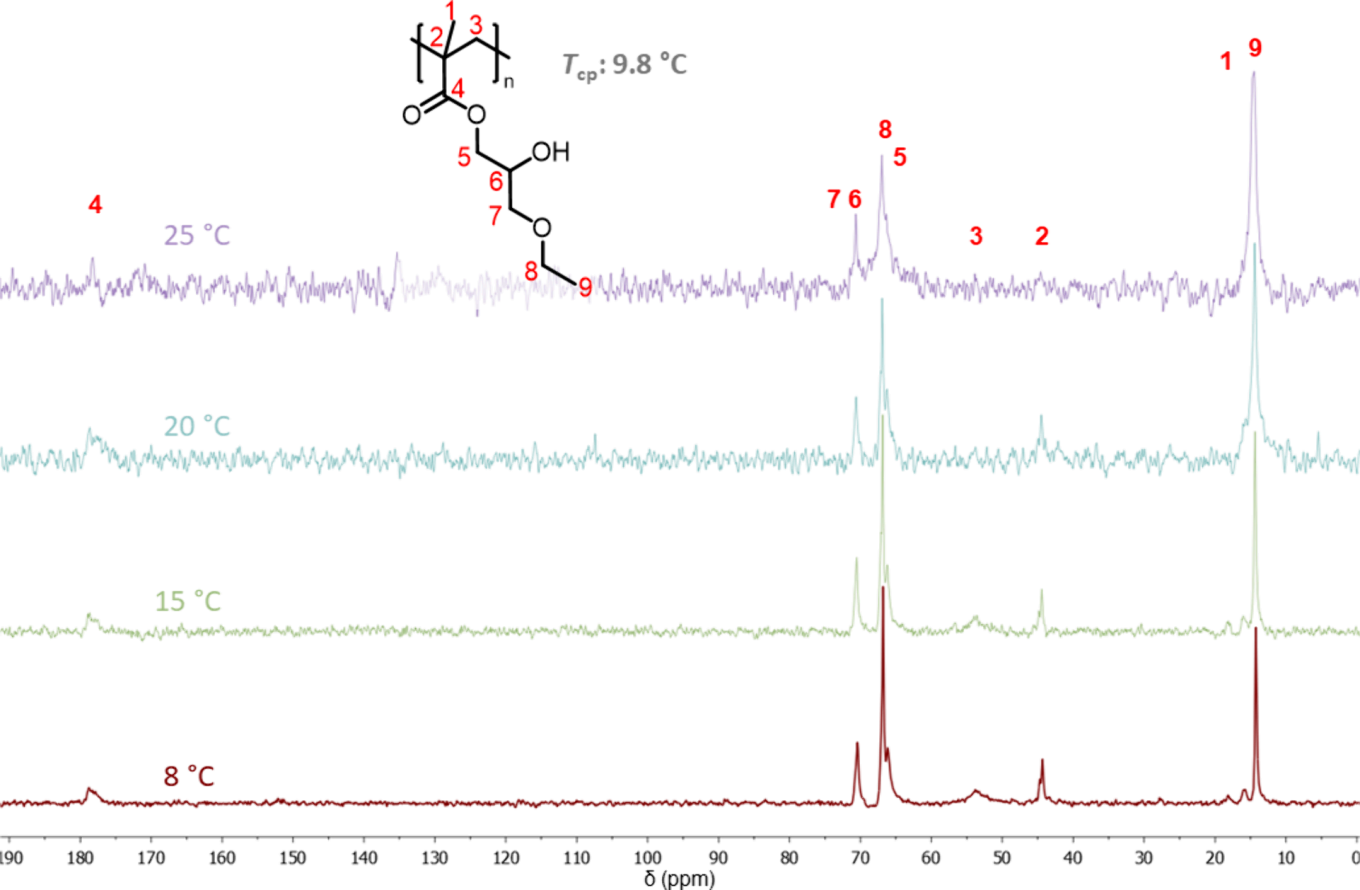
Temperature-dependent ^13^C NMR spectra (D_2_O, 150 MHz) of **pEHPMA** at a concentration of 10 mg mL^–1^ (*T*_cp_: 9.8 °C) acquired after thermal equilibration in a heating cycle. The spectra were referenced to peak **9** at 8 °C. Peaks were assigned with numbers according to the illustrated chemical structure.

In general, the phase transition range of **pEHPA-***b***-BP** is comparable to the one of functional **PGE** coatings at approximately 20 °C and thus well-suited for cell culture applications.^11^**pEHPMA-**based polymers with a lower phase transition range might still be suitable for cell culture, based on literature examples of successful cell detachment at temperatures as low as 4 °C.^20,21^ Furthermore, **pEHPMA-***b***-BP** solutions in PBS showed a slight transmittance increase already at ∼20 °C upon cooling, which might be enough to impact cell behavior, when used as a coating. To investigate the hydration changes of **pEHPMA** on a microscopic scale, temperature-dependent ^1^H and ^13^C NMR spectra were recorded in D_2_O at 10 mg mL^–1^ (Figure S6 and Figure 3).

The ^13^C spectra of the **pEHPMA** solution with a *T*_cp_ of ∼10 °C indicate only minimal chain dehydration on the microscopic scale between 8 and 15 °C, according to minor spectral changes. The transition becomes more pronounced at temperatures of 20 °C and above. For instance, signals **8** and **9** of the outermost carbons of the side chain show marked signal broadening due to reduced mobility upon dehydration and increasing hydrophobic–hydrophobic interactions, particularly between 15 and 20 °C. Furthermore, the declining signal intensity is accompanied by an increasing signal-to-noise ratio, rendering backbone carbon signals **1**+**3** and **2**+**4** undetectable at 20 and 25 °C, respectively. In contrast, the side chain carbon signals **6**, **8** and **9** remain detectable, while signals of **5** and **7** are difficult to differentiate due to similar chemical shifts to signals **6** and **8**. In support of a major loss in chain mobility between 15 and 20 °C, the ^1^H NMR spectra of **pEHPMA** solution (Figure S6) also show a marked decrease in the intensity of all detected signals accompanied with a gradual signal broadening over the whole temperature range, indicating uniform polymer segment dehydration and a continuous phase transition.^44,45^

The observed temperature-dependent dehydration pattern on the microscopic scale across all structural elements is typical for a coil-to-globule transition, as similarly observed with **pNIPAM** or **POX** polymers. However, in contrast to **pNIPAM** and **POX** solutions, which show drastic changes over a 2–3 °C range above their respective macroscopically determined *T*_cp_ values, the microscopic transition process in **pEHPMA** solutions is more continuous.^46,47^ Similar to **pEHPA** polymers, the microscopic dehydration of **pEHPMA** becomes visible in the NMR spectra at temperatures above the *T*_cp_ and proceeds continuously.^3^ However, the dehydration of **pEHPMA** is more pronounced compared to **pEHPA**, as evidenced by the disappearing signals attributed to the backbone carbons and broadened signals of the side chain carbons. Given that the microscopic transition extends up to 25 °C, **pEHPMA**-based coatings might well be suitable for cell sheet detachment at RT. Prior studies indicated that an initiated transition is sufficient for cell sheet detachment through rehydration of the outermost ’fuzzy hair’ layer.^19^ The degree of polymer dehydration upon the thermal phase transition decreases in aqueous **pEHPMA**, **pEHPA**, and virtually nondehydrating poly(hydroxymethoxy methacrylate) (**pHMPMA**)^3^ solutions with the polymer hydrophilicity. The decreasing dehydration is accompanied with a transformation from a coil-to-globule to a liquid–liquid phase separation type. A similar transformation is observed with **NIPAM** copolymers with increasing 2-hydroxyisopropylacrylamide content which exhibit raised *T*_cp_ values and reduced dehydration during the phase transition, correlating with the hydroxyl group content.^48^

### Fabrication and Characterization of Brush Coatings

**Figure 4:**
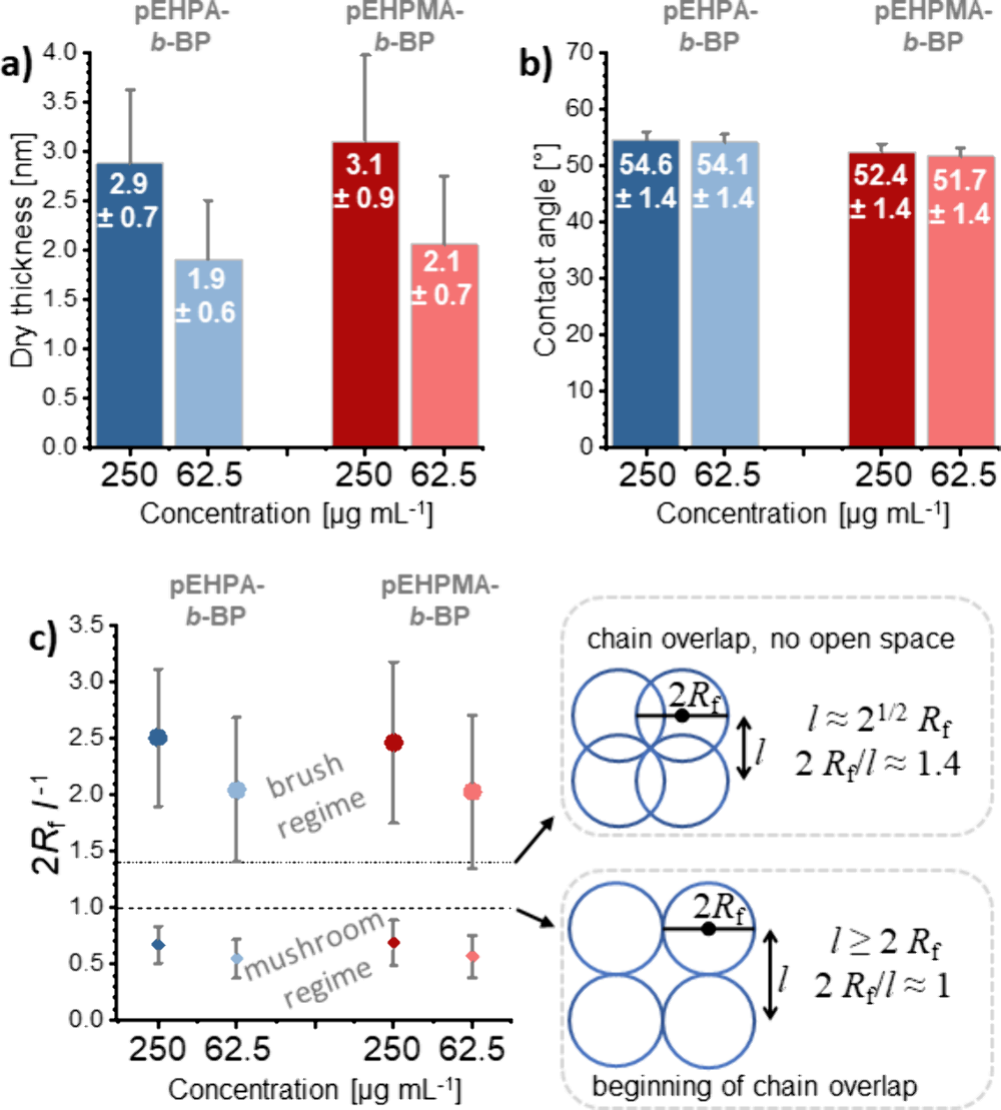
Surface characteristics of **pEHPA-***b***-BP** and **pEHPMA-***b***-BP** brush coatings on PS substrates prepared by adsorption, covalent UV-immobilization, and extraction in ethanol. Concentration-dependent dry layer thicknesses (a), water contact angles (b), and theoretical degree of polymer chain overlap of the grafted polymers in a good (dots) and bad (diamonds) solvent (c). Mean values with SD are plotted (*n* ≥ 4).

Directed adsorption of **pEHPA-***b***-BP** and **pEHPMA-***b***-BP** onto PS surfaces was achieved from dilute water–ethanol mixtures (250 and 62.5 μg mL^–1^) as a selective solvent, based on a previous report.^19,40^ The block copolymer self-assembly parameters were kept identical for both polymers to ensure comparability, as detailed in the experimental section. A sufficient UV-light (360 nm) exposure ensured covalent attachment of the adsorbed polymer brushes on the substrate via C,H-insertion.^49^ Adsorption at 35 °C was established, since preliminary results of **pHMPMA-***b***-BP** immobilization from utilized 62.5 μg mL^–1^ mixtures revealed thicknesses of 1.1–1.8 nm after adsorption at RT and 1.6–2.4 nm after adsorption at 35 °C, including consecutive immobilization and extraction steps (data not shown). To assist the surface adsorption of singularized copolymer chains, their tendency for aggregation (Figure S5) was disturbed by filtering the block copolymer solutions through 0.22 μm syringe filters at 35 °C directly before the self-assembly process. The resulting surface characteristics of the covalently grafted coatings are shown in Figure 4.

For both block copolymers, comparable coating thicknesses of ∼3 and ∼2 nm at concentrations of 250 and 62.5 μg mL^–1^ were obtained, respectively. The directed adsorption of the copolymers *via* their benzophenone block was demonstrated by the significant (*p* < 0.05) differences in dry layer thickness to their respective **pEHPA** and **pEHPMA** homopolymer coatings after adsorption and irradiation but before extraction. The complete thickness loss of the homopolymer layers after extraction (Figure S7a) was detected *via* ellipsometry with slightly reduced CA values of 85–87° (*p* < 0.05) compared to pristine PS (∼90°) (Figure S7b). In contrast, immobilized brush coatings from block copolymer adsorption exhibited reduced contact angle values of 52–55°. In the literature, **pNIPAM** coatings with contact angle values from 38 to 72° have been reported to successfully promote cell adhesion and thermally triggered detachment.^50^ In addition, **PGE** and **pNIPAM** surface coatings with thicknesses of ∼3 nm are suitable for cell sheet fabrication.^9,35^ According to these roughly predictive surface parameters, the produced **pEHPA** and **pEHPMA** would qualify as thermoresponsive substrates for cell culture applications. Additionally, the thermoresponsive behavior of the brush systems between 15 and 39 °C was verified *via* temperature-dependent QCM-D measurements of brush-functionalized PS substrates on QCM-D sensors (Figure S9). **pEHPA-***b***-BP** brush layers revealed a continuous and reversible transition in the scanned temperature range. The transition range of **pEHPMA-***b***-BP** brushes proceeds up to ∼30 °C. The rehydration upon cooling suggests a minor hysteresis. However, this observation needs to be interpreted with caution, due to the generally small ΔΔ*f* differences between PS and **pEHP[M]A-***b***-BP** functionalized surfaces.

To estimate whether the PS substrate is fully covered by the coating during cell culture (37 °C) and detachment (RT) conditions, we calculated the degree of chain overlap (2*R*_f_*l*^–1^) of the surface tethered polymer chains from the ratio of their Flory radius (*R*_f_) to the respective chain anchor distance (*l*) (Table S1). The hydrodynamic radius under cell culture conditions above the polymer’s *T*_cp_ was estimated with a Flory radius in a bad solvent, while at detachment temperatures below the *T*_cp_, the Flory radius in a good solvent was used. These polymer theory-based calculations revealed that the hydrated coatings at temperatures below the polymer’s *T*_cp_ are in the brush regime and transition into a mushroom regime upon dehydration at temperatures above the *T*_cp_ (Figure 4c). Hence, this thermally induced phase transition may expose the basal PS substrate, interfering with proteins and cells. Since our homopolymers nonspecifically interact with the PS surface (Figure S3), full surface coverage by the tethered polymer chains can still be assumed even in a theoretical mushroom configuration under cell culture conditions. This assumption is further supported through AFM images (Figure S8), which reveal uniform coverage and reduced roughness of the coated PS surfaces, when compared to the pristine PS substrate on silicon wafers in dry state.

### Cell Sheet Fabrication and Protein Adsorption

**Figure 5:**
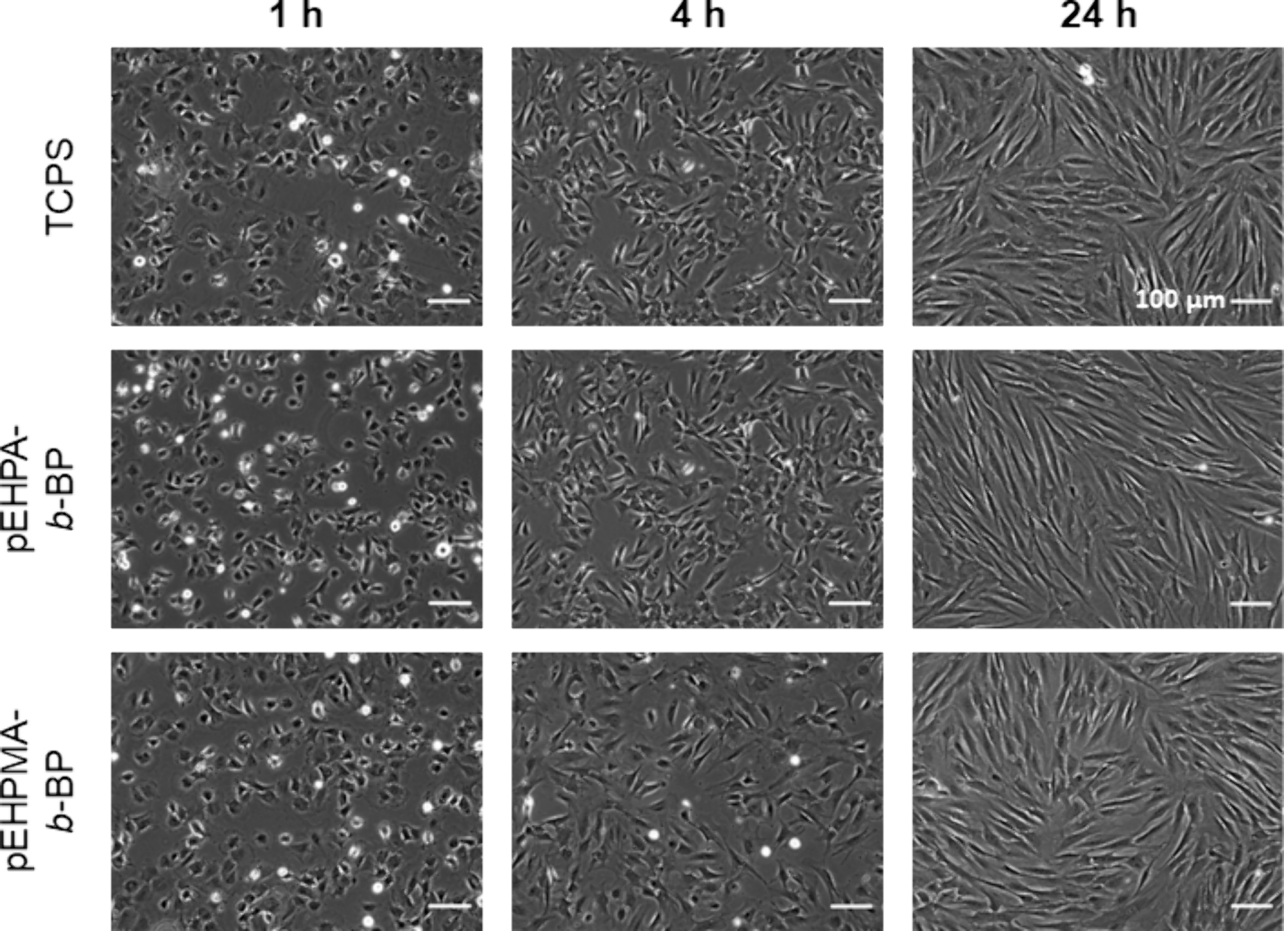
Representative phase contrast images of human dermal fibroblasts on **TCPS** (top), **pEHPA-***b***-BP** (middle), and **pEHPMA-***b***-BP** (bottom) coatings 1, 4, and 24 h after seeding at a density of 43 × 10^5^ cells cm^–2^ (*n* = 3).

The general suitability of **pEHPA-***b***-BP** and **pEHPMA-***b***-BP** coated PS dishes for cell culture was first assessed *via* HDF adhesion and proliferation studies on these substrates and compared to TCPS controls. Therefore, **pEHP[M]A-***b***-BP** coated dishes were sterilized via aq. EtOH (70 v-%) disinfection, as described in the experimental part. Afterward, fibroblasts were seeded at a density of 43 × 10^5^ cells cm^–2^ and were cultured in DMEM supplemented with 10% FBS at 37 °C. Cell morphology was monitored time-dependently after 1, 4, 24, 48, and 72 h via phase contrast microscopy (Figure 5, Figure S10), demonstrating uniform adhesion on all coatings according to the shape change and spreading of fibroblasts on the surface.

Within the first hour, slightly faster cell attachment was observed on TCPS, where 80–90% of the seeded cells were already attached compared to 60–70% on brush coatings. After four hours, ∼ 95% of the cells were attached on all surfaces, and after 24 h, no visible differences in the growing monolayers were observable on the culture substrates. HDF confluency of 100% was reached after 48 h on TCPS and, at the latest, after 72 h on **pEHPA-***b***-BP** and **pEHPMA-***b***-BP** brush coatings.

**Figure 6:**
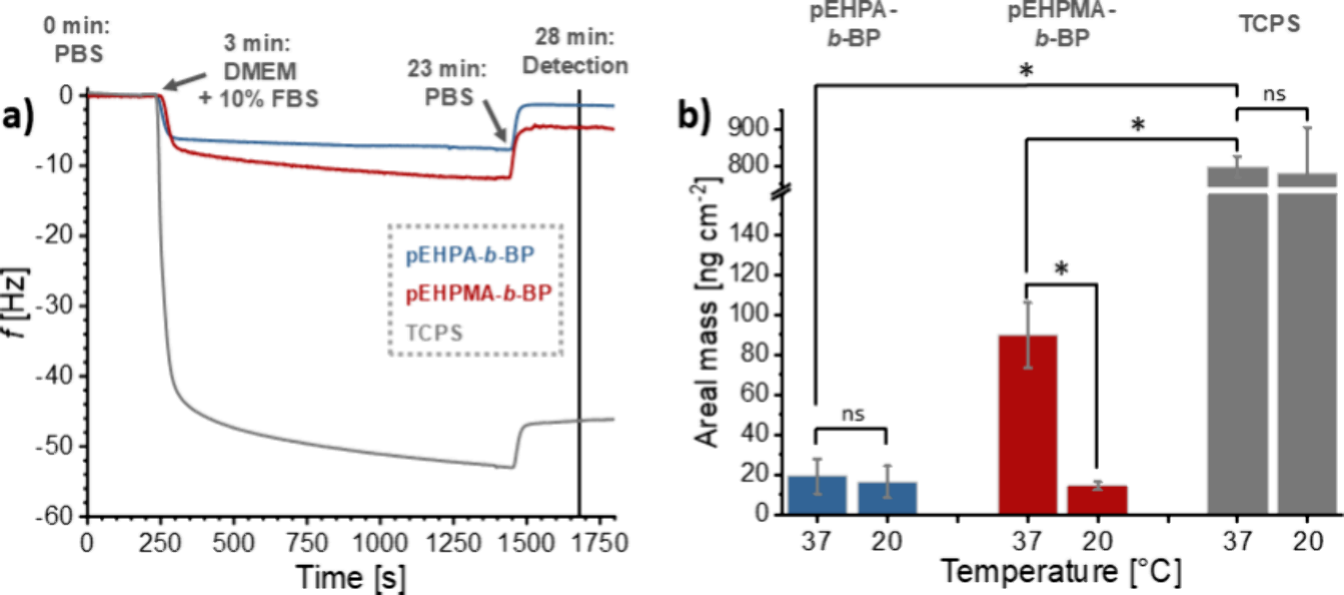
Quantitative protein adsorption from DMEM cell culture medium supplemented with 10% FBS on **pEHPA-***b***-BP** and **pEHPMA-***b***-BP** brush coatings and TCPS controls *via* QCM-D measurements. (a) Representative frequency curves of the protein adsorption at 37 °C. (b) Areal mass of adsorbed proteins derived from the frequency changes Δ*f via* the Sauerbrey equation, measured at the start and end of the experiment, following protein exposure for 20 min and a 5 min PBS flush. Bars indicate mean values along with standard deviation (*n* ≥ 3).

Since cell attachment to the culture substrates is closely linked to protein adsorption, we further screened the initial protein adsorption from DMEM cell culture medium supplemented with 10% FBS medium on the substrates *via* temperature-dependent QCM-D measurements.^13,14^ Representative frequency curves at 37 °C are shown and the calculated areal masses are presented in Figure 6. Additional frequency curves at 20 °C are shown in Figure S10.

For QCM-D measurements, the polymer brushes were prepared on PS-coated QCM-D silicon substrates. Their dry layer thicknesses and contact angles (Figure S11) were comparable to the corresponding coatings on PS-coated silicon wafers. Surprisingly, for **pEHPA-***b***-BP**, no significant difference in adsorbed mass was observed between 37 and 20 °C, with values of 19 ± 9 and 16 ± 8 ng cm^–2^, respectively. The similar values might be attributed to the not yet sufficiently dehydrated state of the **pEHPA** brushes at 37 °C, as detected via temperature-dependent QCM-D measurements (Figure S9) reducing nonspecific, hydrophobic interactions between proteins and the polymer brush. In contrast, **pEHPMA-***b***-BP** brush coatings showed a significant (*p* < 0.1) difference in protein adsorption, with 90 ± 16 ng cm^–2^ at 37 °C and 14 ± 2 ng cm^–2^ at 20 °C, which can be explained by the increased hydrophobicity of pEHPMA brushes in the dehydrated equilibrium above ∼30 °C (Figure S9). However, the adsorbed areal masses on both polymer brushes are an order of magnitude lower than those observed on TCPS (797 ± 29 and 778 ± 125 ng cm^–2^ at 37 and 20 °C, respectively). Low protein adsorption below the polymers’ *T*_cp_ is characteristic of thermoresponsive coatings that facilitate thermally induced cell detachment.^11,28^ In contrast, efficient cell adhesion under cell culture conditions typically requires higher amounts of adsorbed proteins. Thus, the low amounts of adsorbed proteins on the fully synthetic **pEHPA-***b***-BP** and **pEHPMA-***b***-BP** brushes at 37 °C appear contradictory to the observed cell adhesion and proliferation on these substrates (Figure 5), as cell adhesion is generally a protein-mediated process.

Aligning with this theory, thermoresponsive **PGE** coatings adsorb protein areal masses between 400 and 700 ng cm^–2^ from FBS-containing cell culture media at 37 °C.^11,19^ The distinct adsorption values vary with the **PGE** copolymer composition and substrate type. Fn, one of FBS’s main cell adhesive proteins, has been the focus of several single-protein adsorption studies. These studies demonstrate reduced areal masses of adsorbed Fn compared to the adsorbed protein masses from multiprotein mixtures like FBS. For instance, thermoresponsive **pNIPAM** hydrogel coatings (∼16 nm) adsorb only ∼150 ng cm^–2^ Fn at 37 °C, with no Fn adsorption detected at 20 °C.^28^ Similarly, poly(*n*-propyl oxazoline) (**P***n***PrOX**) bottlebrushes (∼270 nm) adsorb an areal mass of 90 ng cm^–2^ Fn at 37 °C, enabling cell adhesion and proliferation on Fn pretreated substrates.^12,51^

Besides the adsorbed amount, the activity of Fn in presenting functional RGD units is crucial for recognition by anchorage-dependent cells. Protein-resistant, nonresponsive **pHEMA** brushes,^52^ which comprise a methacrylic backbone and β-hydroxy side chains similar to **pEHPA** and **pEHPMA**, adsorb only ∼4–6 nm cm^–2^ Fn on 3–20 nm thick coatings from FCS-containing cell culture medium.^53^ Interestingly, the RGD activity decreased abruptly between coatings of 3 and 6 nm thickness, reducing the spreading and increasing the migration of cultured vascular smooth muscle cells. The high RGD activity on 3 nm thick brushes was explained through the limited penetration depth of adsorbed Fn on a thin brush, resulting in unmasked RGD domains.^53^ A similarly active Fn layer adsorbed from FBS-containing medium at 37 °C could explain the observed cell adhesion on thin **pEHP[M]A-***b***-BP** coatings in this study, despite the comparably low areal mass of adsorbed proteins. The reorientation of the thermoresponsive polymer brush during the thermally triggered rehydration, in turn, initiates and contributes to the detachment of the cell sheet.

**Figure 7:**
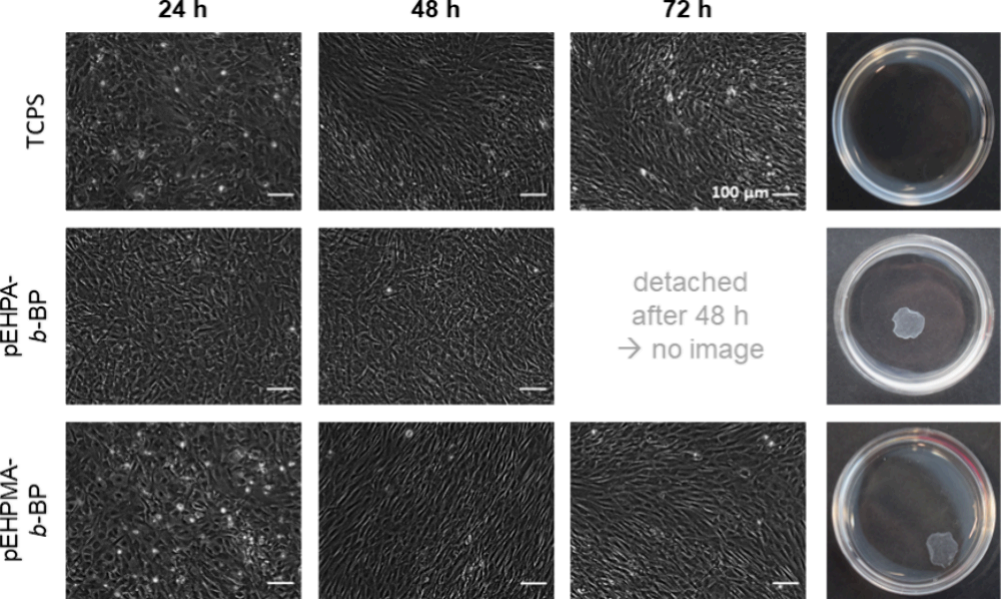
Representative phase contrast images of human dermal fibroblasts on **TCPS** (top), **pEHPA-***b***-BP** (middle), and **pEHPMA-***b***-BP** (bottom) coatings 24, 48, and 72 h after seeding at a density of 104 × 10^5^ cells cm^–2^ and photographs of the resulting cell sheets after the thermally triggered detachment procedure (*t*_max_ (**pEHPA-***b***-BP**) = 60 min, *t*_max_ (**pEHPMA-***b***-BP**) = 120 min). Cells on TCPS controls did not detach under the same conditions (*n* = 4).

Additional thermally triggered cell detachment experiments were performed with confluent cell monolayers produced from an initial HDF seeding density of 104 × 10^5^ cm^–2^. Representative phase contrast images after 24, 48, and 72 h, along with macroscopic photographs of detached sheets, are shown in Figure 7.

HDFs on TCPS and **pEHPA-***b***-BP** surfaces reached confluency after 48 h, and the detachment procedure reproducibly yielded fully detached cell sheets within one hour. In some cases, initiated detachment was already observed after the first exchange of warm medium to PBS at RT, with complete detachment after a 10 min incubation at 37 °C. To ensure the viability of the thermally detached cells from **pEHPA-***b***-BP** coatings, cell sheets were trypsinized, stained with propidium iodide, and counted *via* flow cytometry alongside controls cultured on TCPS and harvested by conventional trypsinization (Figure S13). No significant difference in the percentage of dead cells was observed within the standard deviation (**pEHPA-***b***-BP**: 1.7 ± 0.2%, **TCPS**: 1.5 ± 0.1%, n = 3), demonstrating the general cell compatibility of the newly developed **pEHPA-***b***-BP** coatings.

In contrast, thermally triggered cell detachment from **pEHPMA-***b***-BP** surfaces was less efficient. Confluency was achieved only after 72 h, and reliable initiation of the detachment process required incubation at 4 °C. Additionally, cells did not always detach as a complete sheet, sometimes resulting in sheets with holes or fragmentation. Comparatively, **pEHPA-***b***-BP** coatings performed more reliably without sheet disruption and were thus selected for subsequent sterilization studies with on-par performing **PGE** brush coatings.^11^

### Sterilization of Brush-Coated Surfaces by FO Gas and Gamma Radiation

Sterile ready-to-use culture ware is essential in routine cell culture and for biomedical products, ensuring product safety and performance reliability. Noninvasive gas and radiation procedures enable industrially normed sterilization of substrates directly in their final packaging, making them ideal for upscaling and translation efforts. However, the oxidative and radical fragmentation mechanisms that neutralize biological entities and pathogens can also adversely affect the functional materials by chemical fragmentation or cross-linking, as observed with **PEG**, **pNIPAM**, and **POX**.^34,37,54^

**Figure 8:**
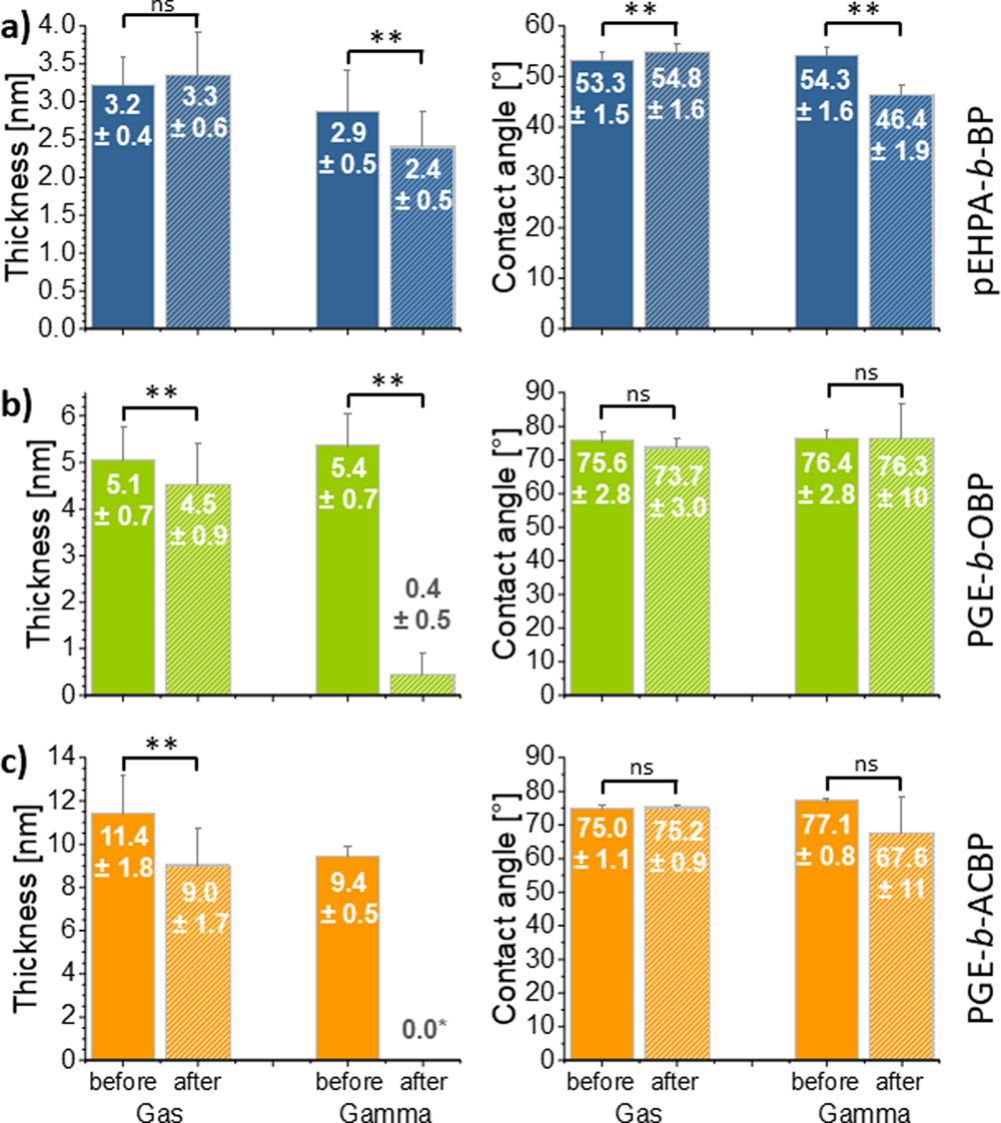
Dry layer thicknesses and water contact angles before and after sterilization by FO gas and gamma treatment with subsequent extraction of the **pEHPA-***b***-BP** (a), **PGE-***b***-OBP** (b), and **PGE-***b***-ACBP** (c) brush-coated substrates. Number values and bars indicate mean values with standard deviation (*n* ≥ 3). *All measured values were below the basal TCPS layer, resulting in negative thickness values indicative of oxidative degradation of not only the brush coating but also the basal substrate.

To assess the stability of the functional thermoresponsive surfaces developed in our previous and current work, we evaluated their surface properties and performance in cell sheet fabrication after sterilization. Therefore, **PGE-***b***-OBP** brushes on PS, **PGE-***b***-ACBP** brushes on TCPS, and **pEHPA-***b***-BP** brushes on PS were prepared on silicon wafers and evaluated after sterilization by treatment with FO gas or gamma radiation normed to DIN standards. The sterilized model substrates were extracted for 16 h in ethanol to remove any degraded surface components and assess the coating stability. To consider the impact of storage time, the nonsterilized brush coatings’ dry layer thickness and CA values were evaluated after storage at ambient conditions for 14–18 d, resembling the average time between sample preparation and analysis after sterilization treatment. An overview of these substrate parameters before and after sterilization, including extraction, is shown in Figure 8. Corresponding results for the sole impact of storage time are shown in Figure S14.

The **pEHPA-***b***-BP** brushes were preserved on the PS-coated silicon wafers after FO and gamma sterilization, as shown in Figure 8a. While thickness values did not change significantly after FO sterilization, a slight increase in contact angle (*p* < 0.05) from ∼53 to ∼55° indicates a possible surface reaction with the FO gas or coating reorientation during the procedure. The latter seems more likely, since no significant difference was found between CA values of FO-treated surfaces, CA values of untreated **pEHPA-***b***-BP** brushes in Figure 4b, and only 14–18 d stored **pEHPA-***b***-BP** samples (Figure S14). In contrast, gamma sterilization significantly decreased the dry layer thickness of the brush from 2.9 to 2.4 nm and the CA value from 54.3° to 46.4°, suggesting possible oxidation and/or partial degradation or cross-linking of the coatings. Gamma or e-beam radiation in the presence of ambient oxygen have been shown to oxidize hydroxyl groups, cleave ester bonds and induce cross-linking reactions, which in turn can increase surface hydrophilicity, limit chain movement and reduce the layer thickness.^34,39^

Similar effects but to a larger extent were observed for the **PGE** brush systems, drastically degrading the polyether coating by gamma sterilization. After radiative gamma treatment and surface extraction, the **PGE-***b***-OBP** brush thickness was reduced to ∼0.4 nm. Furthermore, the CA values varied more drastically from 76.4 ± 2.8° before to 76.3 ± 10° after the treatment, with individual measurements ranging from 86° to 60°. In contrast, FO treatment resulted in a minor decline of the dry thickness from 5.1 to 4.5 nm, which might simply be attributed to an effect of storage time (Figure S14b), indicating no distinct impact of FO sterilization. Furthermore, nonsignificant CA changes from ∼75.5° to 73.7° were observed.

The **PGE-***b***-ACBP** brush thickness declined from 9.5 to 5.3 nm under ambient storage conditions, which we attribute to a drying effect of the polymer layer during long-term dry storage. The FO gas sterilized surfaces showed a similar decline from 11.4 nm before to 9.0 nm after the treatment, with no significant alterations of the CA regardless of the gas treatment (Figure 8c) or ambient storage for 14–18 d (Figure S14c). As expected, also the **PGE-***b***-ACBP** coating on TCPS substrates was drastically degraded by gamma sterilization, resulting in even negative thickness values indicative of additional degradation of the basal TCPS layer. Accordingly, the CA value declined from 77.1 ± 0.8° to 66.5 ± 10°, suggesting a nonuniform surface state. Control experiments on uncoated PS substrates revealed that gamma sterilization also significantly impacts the pristine PS surface by lowering CA values from 89.5° to 74.7° (Figure S15). FO sterilization only had a minimal impact on PS surfaces, with a resulting contact angle of 87.7°, as shown in Figure S15.

**Figure 9:**
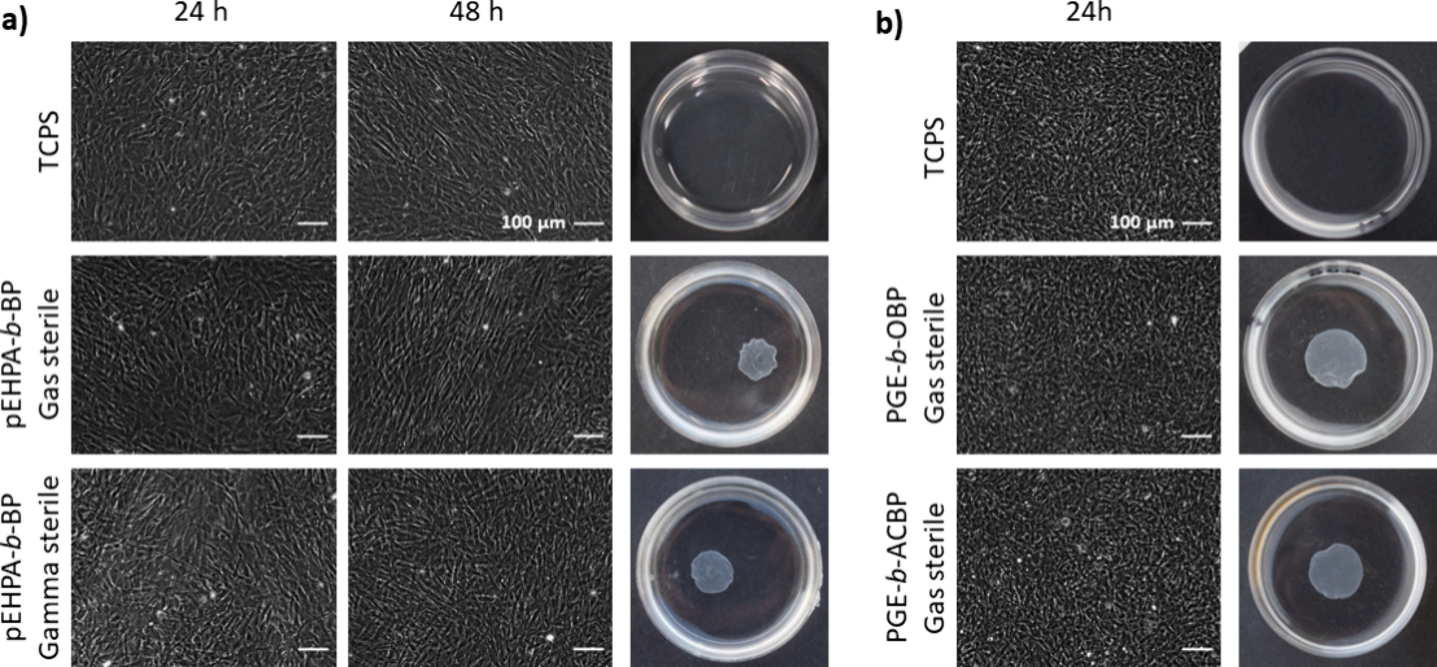
Representative time-dependent phase contrast images of HDFs cultures on sterilized substrates and photographs of resulting cell sheets after thermally triggered detachment from thermoresponsive coatings (*n* ≥ 3). (a) TCPS controls (top, no detachment) and **pEHPA-***b***-BP** brush-coated PS substrates after FO (middle, *t*_max_ = 90 min) and gamma (bottom, *t*_max_ = 16 h) sterilization with a seeding density of 104 × 10^5^ cells cm^–2^. (b) TCPS controls (top, no detachment) and FO-sterilized **PGE-***b***-OBP** (middle, *t*_max_ = 180 min) and **PGE-***b***-ACBP** (bottom, *t*_max_ = 180 min) brush-coated substrates with a seeding density of 160 × 10^5^ cells cm^–2^.

To further evaluate the impact of the sterilization treatment on the functionality of the thermoresponsive coatings in cell culture, the brush-coated cell culture dishes were employed in cell sheet detachment experiments under previously established cell culture conditions. Based on the results shown in Figure 8, we excluded gamma sterilized **PGE** surfaces. Representative phase contrast images of HDFs cultured on the sterilized brush coatings with TCPS controls are in Figure 9 along with macroscopic photographs of the dishes after exposure to detachment conditions.

HDFs adhered and proliferated comparably on sterilized and aq. EtOH (70 v-%) disinfected coatings, confirming the cell attachment-supporting nature of all coatings. Moreover, also the thermally triggered detachment of HDF cell sheets after FO sterilization was successful on all tested **PGE** and **pEHPA-***b***-BP** coated substrates employing the established detachment conditions. Thus, we can conclude that the normed FO gas treatment for effective sterilization is compatible with the developed functional brush coatings (n = 3). However, sheet detachment on **PGE** coatings was slowed, with timeframes reaching up to 180 min, compared to ∼60 min for freshly prepared and disinfected (70% aq. EtOH) coatings.^11^ Detachment times on **pEHPA-***b***-BP** coatings ranged from 20 to 90 min, slightly exceeding the maximal time frame of 60 min determined with disinfected brush surfaces. These results on the functional performance of the coatings align with the observed surface changes of **PGE** and **pEHPA-***b***-BP** coatings after storage and/or FO treatment. More pronounced loss in dry layer thickness through storage or FO treatment of **PGE** brushes, even if only caused by drying effects, correlates with impaired functionality in thermally triggered cell sheet detachment but not in cell adhesion and proliferation.

Gamma sterilization significantly impaired the functionality of the **pEHPA-***b***-BP** coatings as indicated by the markedly altered surface properties. While there was no noticeable impact on the cell adhesion and culture, the established detachment protocol, exchanging the warm medium with PBS at RT, did not induce cell detachment (n = 3). Therefore, we adopted the procedure established for **pEHPMA-***b***-BP** coatings, which helped to induce the detachment, but required up to ∼16 h, thus exceeding a useful application time frame due to possible cell apoptosis after 4 or more hours.^55^ Additionally, in ∼50% of the experiments (n = 7) the cell sheets did not fully detach, indicating a true loss of functionality on gamma sterilized **pEHPA-***b***-BP** surfaces.

Overall, the obtained results demonstrate the benefit of the newly developed thermoresponsive coatings with an aliphatic backbone in terms of coating stability and functional performance in cell culture. While gas-sterilized **PGE** brushes exhibited prolonged cell sheet detachment times after 14–18 d of dry storage, detachment times of gas-sterilized **pEHPA-***b***-BP** surfaces remained almost unaffected, with minimal changes in surface properties. These findings align with reports on ethylene oxide gas-sterilized **PEG** hydrogels and surfaces, which retain the morphological properties or coating thickness but can exhibit slightly altered cross-linking density.^37,39^ Ultimately, oxidation through ambient oxygen can limit the air storage stability of **PEG** in bulk to less than a month.^38,56^ The stability of **pEHPA-***b***-BP** coatings appears comparable to **pNIPAM** coatings, which can also be gas sterilized without adverse effects on structure and properties.^33^

Gamma irradiation significantly damaged all evaluated surface coatings. Similarly, previous reports indicate strong morphological changes and an increase in free radicals and cross-linking in radiation treated **PEG** hydrogels.^37,57^ Free radicals can lead to chain scission and degradation, explaining the observed drastic loss in thickness. γ Radiation of **pNIPAM** solutions with a comparable dosage (50 kGy) resulted in an increased LCST and hampered transition, along with increased molecular weight and polydispersity due to cross-linking reactions.^34^ Furthermore, controlled oxidation of hydroxyl groups to carbonyl groups in **pHEMA** brush systems leads to a bioadhesive system, which cannot be excluded for **pEHPA-***b***-BP** brushes.^58^ Additionally, also the underlying PS substrate can get oxidized, as indicated in Figure S15, decreasing the overall thickness and rendering **pEHPA-***b***-BP** brush systems partially nonresponsive, which causes the loss of functionality. Nevertheless, gas sterilization remains an attractive option for a scalable and effective sterilization process when aiming at transfer of such functional coatings from research to market.

## Conclusions

Thermoresponsive brush coatings, based on **pEHPA-***b***-BP** and **pEHPMA-***b***-BP** polymers, with adjustable dry layer thicknesses up to ∼3 nm were successfully immobilized on PS surfaces for cell sheet fabrication. Despite unusually low protein adsorption at 37 °C (∼20–90 ng cm^–2^), both coatings demonstrated cell adhesive properties, likely due to a thin but active layer of fibronectin, as observed on structurally similar **pHEMA** coatings.^53^**pEHPA-***b***-BP** coatings, with a *T*_cp_ of 20.3 °C, reliably detached fibroblast sheets after cooling to RT. In contrast, **pEHPMA-***b***-BP** coatings, with a *T*_cp_ of 6 °C, required cooling to 4 °C, occasionally resulting in perforated or disrupted cell sheets. The stability of **pEHPA-***b***-BP** and previously established **PGE** coatings on PS and TCPS toward sterilization and short-term storage (up to 18 d) was further evaluated. Gamma sterilization with a dose of ∼45 kGy was found to be too harsh, completely degrading **PGE** and adversely affecting **pEHPA-***b***-BP** coatings, as evidenced by decreasing thickness and CA values, along with significantly hampered cell sheet detachment. In contrast, gas sterilization had no significant impact on **pEHPA-***b***-BP** coatings, although detachment times slightly increased in some cases. The stability of **pEHPA-***b***-BP** coatings during storage and after gas sterilization highlights the advantage of an aliphatic backbone system since these coatings maintained their functionality better than **PGE** coatings. Future work will explore the compatibility of these coatings with different cell types. Experiments toward an understanding of the underlying mechanisms of minimal protein adsorption, which impressively mediate cell adhesion, are in progress.

## Supporting Information Available

Materials and methods, monomer and polymer synthesis and characterization, additional temperature-dependent turbidimetry and DLS data of utilized polymers, temperature-dependent ^1^H NMR spectra of pEHPMA in D_2_O, additional thickness and CA values of **pEHPA** and **pEHPMA** adsorption experiments, calculation of chain overlap parameters, AFM images, temperature-dependent phase transition of brush layers probed via QCM-D measurements, additional cell culture images and live dead staining data, properties of prepared QCM-D chip surfaces, thickness and CA values for storage-only surfaces, and CA evaluation of sterilized PS surfaces (PDF)
