# Supplementary material for: Thermoresponsive Brush Coatings for Cell Sheet Engineering with Low Protein Adsorption above the Polymers’ Phase Transition Temperature
Source: ACS Appl Bio Mater. 2024 Nov 5;7(11):7544–55. doi: 10.1021/acsabm.4c01127 (PMC11577427; doi:10.1021/acsabm.4c01127)
Supplement: Supplementary file 1 — mt4c01127_si_001.pdf [file mt4c01127_si_001.pdf]

Supporting Information for the following manuscript

Thermoresponsive brush coatings for cell sheet engineering with low protein adsorption above the polymers' phase transition temperature

Alexander Schweigerdt[1], *Daniel D. Stöbener*[1,2], *Johanna Scholz*[1], *Andreas Schäfer*[1], *Marie Weinhart*[1,2]*

[1] Institute of Chemistry and Biochemistry, Freie Universitaet Berlin, Takustr. 3, 14195 Berlin, Germany

[2] Institute of Physical Chemistry and Electrochemistry, Leibniz Universitaet Hannover, Callinstr. 3A, 30167 Hannover, Germany

* Corresponding author: email marie.weinhart@pci.uni-hannover.de and marie.weinhart@fu-berlin.de, phone: +49 511-762 14938

Content

1. **Materials and Methods** 4

1.1. **Materials** 4

1.1.1. **Materials for cell culture** 5

1.2. **Methods** 5

1.2.1. **Preparation of model substrates** 7

1.2.2. **Cell Isolation and Culture** 7

2. **Monomer synthesis** 8

2.1. **Synthesis of EHPMA** 9

2.2. **Synthesis of BPA** 10

2.3. **Synthesis of BPMA** 10

3. **Polymer synthesis** 11

3.1. **Synthesis of pEHPMA** 12

3.2. **Synthesis of pEHPMA-*b*-BP** 14

3.3. **Synthesis of pEHPA-*b*-BP** 15

3.4. **Calculation of the benzophenone repeating units from the ^{1}H NMR spectra** 16

4. **Sample preparation and analysis of the thermoresponsive behavior** 17

4.1. **Turbidimetry** 17

4.2. **DLS measurements and intensity distribution of polymer samples** 19

4.3. **Temperature-dependent pEHPMA ^{1}H NMR measurements** 20

5. Comparison of homo- and block copolymer adsorption on PS 22

5.1. Estimation of the chain overlap parameters based on polymer theory 23

6. Surface topography and thermoresponsive behavior of brushes on PS surface 24

7. Additional cell adhesion images 27

8. Surface parameters of brush-coated QCM-D chips 28

9. Viability of thermally detached cells..30

10. Storage stability of coated substrates 31

11. Impact of gamma and FO treatment on CAs of pristine PS substrates 32

12. References 33

1. Materials and Methods

1.1. Materials

All materials and chemicals were used as received unless stated otherwise. Acryloyl chloride, methacryloyl chloride and deuterated chloroform ($CDCl_3$) were purchased from ABCR (Karlsruhe, Germany). Glycidol, boron trifluoride etherate ($BF_3 \cdot OEt_2$, 46% solution in diethyl ether), azobis(4-cyanovaleric acid) (ACVA), dimethyl formamide (DMF) and 4-cyano-4-(phenylcarbonothioylthio)pentanoic acid (radical addition fragmentation transfer (RAFT) agent) were purchased from Sigma Aldrich (Steinheim, Germany). Triethylamine (NEt_3) was purchased from Sigma Aldrich or TCI GmbH (Eschborn, Germany). Glycidyl methacrylate and 4-hydroxybenzophenone were purchased from TCI GmbH. 4-Dimethylaminopyridine (DMAP) was purchased from Carbolution chemicals (St. Ingbert, Germany). Dichloromethane (DCM), diethyl ether, methanol, acetonitrile, Al_2O_3, and $NaHCO_3$ were purchased from Fisher Chemical (Loughborough, UK). Ethanol and ethyl acetate (EA) were purchased from Merck (Darmstadt, Germany). Cyclohexane (CH), Na_2SO_4, deuterated methanol-d4 (MeOD), and deuterated water (D_2O) were purchased from Carl Roth (Karlsruhe, Germany). 1,4-Dioxane and $MgSO_4$ were purchased from Grüssing (Filsburg, Germany).

Deionized water with a minimum resistivity of 18.0 MΩ cm was used (Millipore™). Dulbecco’s phosphate-buffered saline (PBS) was purchased from Thermofisher (Henningsdorf, Germany). Pre-wetted regenerated cellulose dialysis tubes (molecular weight cut-off: 3.5 kDa, Spectra/Por® 6) from Spectrumlabs were used for dialysis. Silicon wafers were supplied by Silchem GmbH (Freiberg, Germany). QCM-D QSXT 303 chips with silicon surface were purchased from Biolin Scientific (Gothenburg, Sweden).

1.1.1. Materials for cell culture

Falcon® PS culture dishes (35 mm diameter) were purchased from Th. Geyer GmbH & Co. KG (Berlin, Germany) and Corning® tissue culture PS (TCPS, 35 mm diameter) were supplied by VWR International (Leuven, Belgium). Dulbecco's modified Eagle medium (DMEM) was purchased from Thermo Fisher Scientific (Darmstadt, Germany). Propidium iodide (PI) was supplied by Sigma Aldrich (Steinheim, Germany). Fetal bovine serum (FBS, #S0115) was purchased from Biochrom GmbH (Berlin, Germany). All other expendable materials for cell culture were purchased from Sarstedt (Nümbrecht, Germany).

1.2. Methods

Column chromatography was performed on a Teledyne Isco CombiFlash Rf+ (USA) using RediSep NP80 columns and HPLC-grade cyclohexane and ethyl acetate. Polymer dialysis was performed against methanol (1.8 L) for 48-72 hours, and the solvent was exchanged at least three times. Gel permeation chromatography (GPC) in tetrahydrofuran (THF) as eluent was conducted on an Agilent 1100 Series instrument with concentrations of 3.5 mg mL^{-1} and a flow rate of 1 mL min^{-1} at 25 °C. Three PLgel 5 µm mix-C columns with the dimensions 7.5 × 300 mm from Agilent (Waldbronn, Germany) were used in line with a refractive index detector and polystyrene (PS) standards from PSS (Mainz, Germany) were used for calibration. Intensity-dependent size distributions were measured on a Malvern Zetasizer Ultra analyzer (Malvern Instruments) using a He−Ne laser (λ = 633 nm) and scattering detection at 173°. Aqueous polymer solutions were equilibrated for 120 s at the corresponding temperature before each measurement. Nuclear magnetic resonance (NMR) spectra were processed with the MestreNova 10.0.2 software, referenced to the solvent peak of the deuterated solvent unless stated otherwise, and the chemical

shifts δ were reported in ppm. Spin-coating was performed using a WS-650-23 spin-coater from Laurell Technologies Corporation (North Wales, PA, USA). Photoimmobilization of brush coatings was performed with ultraviolet (UV)-light using a UV-KUB 2 from KLOÉ (Montpellier, France) with a wavelength of 365 nm and a radiant exposure of 4.0 J cm^{-2}. Spectroscopic ellipsometry (SE) was performed on a SENpro ellipsometer and evaluated with SpectraRay 3 (Version 5.3.2.1853) software from SENTECH instruments GmbH (Berlin, Germany). Static water contact angles (CA) were measured with an OCA contact angle system from DataPhysics Instruments GmbH (Filderstadt, Germany) and fitted with the software package SCA202 (version 3.12.11). The nano-structure of the PS wafer substrates and applied brush coatings was analyzed by atomic force microscopy (AFM) using a NanoWizard IV AFM (JPK Instruments, Berlin, Germany). The AFM head was equipped with a 15 µm z-range linearized piezoelectric scanner and an infrared laser. Imaging of the samples was performed in air in tapping mode using Tap300Al-G silicon sensors with a tip radius of approximately 10 nm and a nominal resonant frequency 200 – 400 kHz (280-290 kHz for the measurements). Imaging parameters were adjusted to minimize the force applied on surfaces. Acquisition of 2 × 2 µm images was optimized to 2 Hz line speed and 256 × 256 acquisition points. Imaging data were analysed with the JPK image processing v.6.4.21 (JPK Instruments, Berlin, Germany). QCM-D measurements were performed on a Q-Sense E1 system from LOT QuantumDesign (Wertheim, Germany) with a standard flow module and a Reglo Digital peristaltic pump from Ismatec (Wertheim, Germany). Polystyrene-coated (25-30 nm) silicon QCM-D sensors were used for the experiments. QSoft401 software (version 2.5.22) was used for data acquisition and QTools 3 software (version 3.1.25) from Biolin Scientific AB 2000-2014 (Stockholm, Sweden) was used for data analysis. The temperature-dependent phase transition behavior of the brush coatings was observed in a quasi-static mode by measurements in Milli-Q water at 0.01 mL min^{-1} flow. After sensor equilibration for a minimum

of 30 min at 15 °C the temperature was incrementally increased and subsequently decreased in 3 °C steps (15-18-21-23-25-27-30-33-36-39 °C) with a heating/cooling rate of 1 °C min^{-1}. Each temperature step was followed by a 30 min equilibration period. Frequency (Δf) changes of the 3rd harmonic at the end of each equilibration period were used for evaluation. The fundamental resonance frequency usually suffers from poor energy trapping as well as poor reproducibility and was therefore discarded.[1, 2] Microscopic images were taken on a Zeiss Observer Z1 from Carl Zeiss Microscopy GmbH (Jena, Germany) and evaluated with the software Zen 2 Version 2.0.0.0 from Carl Zeiss Microscopy GmbH (Jena, Germany). Macroscopic photographs were taken with a Nikon D3100 from Nikon GmbH (Düsseldorf, Germany). Flow cytometry was performed on Attune NxT cytometer from Thermo Fisher.

1.2.1. Preparation of model substrates

PS model substrates were prepared by spin coating silicon wafers (11 × 11 mm) or QCM-D chips using a 1-2% (w/w) solution of PS (35 µL) in toluene at 3000 rpm for 60 s, resulting in PS thickness values of 30-80 nm. PS solutions were prepared using PS (M_n = 132 kDa, Đ = 1.9) from Falcon® culture dishes supplied by Th. Geyer GmbH & Co. KG (Berlin, Germany). Tissue culture polystyrene (TCPS) substrates were fabricated by irradiation of PS coated silicon wafers in a Beltron UV chamber (Hg lamp, 850 W) from Beltron GmbH (Rödermark/Urberach, Germany) at a distance of 24 cm (~10 mW cm^{-2}) for 150 s. The oxidized samples were extracted in ethanol for 1 d, subsequently washed with Milli-Q water and directly used for coating experiments within 1–2 d.

1.2.2. Cell Isolation and Culture

Cell isolation and culture was performed analogously to previously established protocols.[3] Human dermal fibroblasts (HDF) were isolated from human foreskin biopsies after ethical approval and

informed parental consent. Connective tissue was mechanically removed, and remaining skin tissue was dissected into approximately 1 × 10 mm stripes. They were incubated in dispase II (2 U mL^{-1} in PBS) for 16 h at 4 °C. Epidermis and dermis were mechanically separated, followed by mincing of the dermis. Minced tissue was digested with collagenase NB4 (0.5 U mL^{-1} in PBS with Ca^{2+} and Mg^{2+}) for 45 min at 37 °C. After centrifugation (200 × g, 5 min), the pellet was washed with culture medium consisting of DMEM supplemented with 10% FBS, 100 U mL^{-1} penicillin, and 100 µg mL^{-1} streptomycin. The tissue fragments were resuspended in 2 mL of medium and seeded into a 75 cm^2 tissue culture flask. Additional 6 mL medium was added to the outgrowing cells after 24 h. Cells were cultured in a humidified atmosphere at 37 °C and 5% CO_2 and used in passages 3 to 8.

2. Monomer synthesis

The synthesis of 2-hydroxy-3-ethoxypropylacrylate (**EHPA**) was performed according to previous report.[4] The synthesis of further monomers 2-hydroxy-3-ethoxypropylmethacrylate (**EHPMA**), benzophenone acrylate (**BPA**), and benzophenone methacrylate (**BPMA**) was performed based on

literature procedures.[4, 5] The reaction pathways for **EHPMA**, **BPA**, and **BPMA** are shown in Scheme S1.

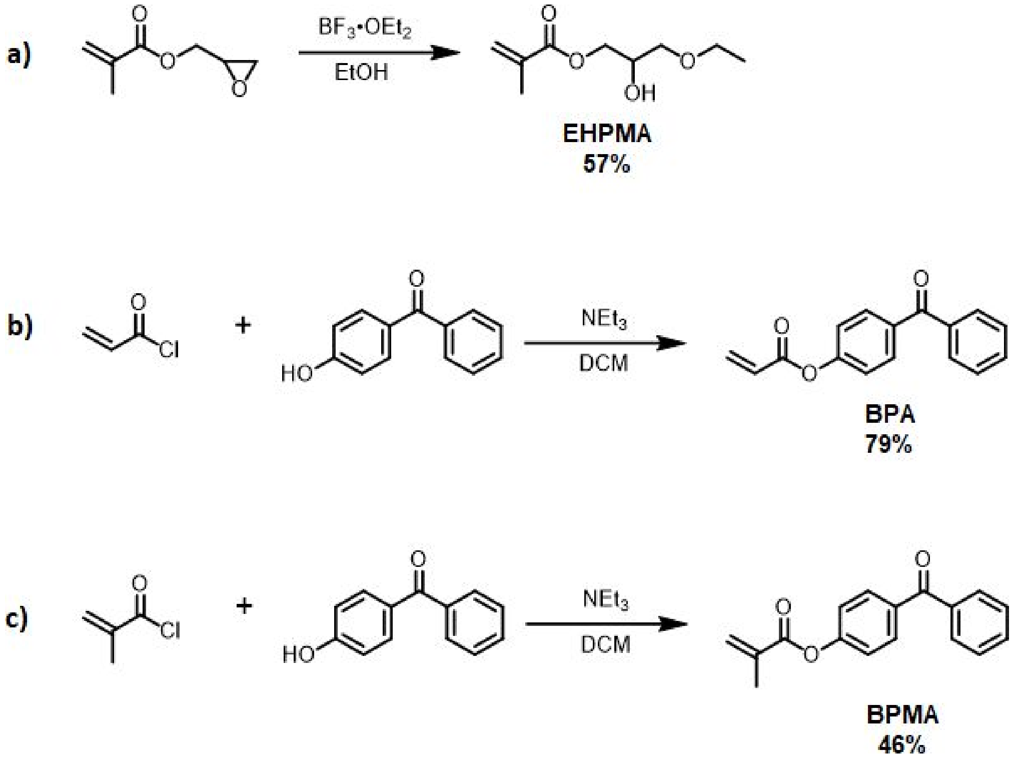

Scheme S1. General synthesis route for the **EHPMA** (a), **BPA** (b), and **BPMA** (c) monomers with isolated yields after purification.

2.1. Synthesis of EHPMA

$BF_3 \cdot OEt_2$ (0.18 mL, 1.41 mmol, 0.1 eq) was dissolved in ethanol (18.5 mL, 317 mmol, 22 eq) and the mixture was heated to 50 °C. Glycidyl methacrylate (1.95 ml, 14.3 mmol, 1 eq) was added dropwise over 10 min. The mixture was stirred until no starting material could be detected via thin-layer chromatography (1.5 h). The reaction mixture was cooled down to 0 °C and 20 mL of THF was added. The reaction mixture was filtered over an Al_2O_3 plug, washed with 1/1 v:v THF/EtOH mixture (4 × 20 mL), and concentrated under reduced pressure. Purification via flash chromatography (CH:EA 7/3 v:v) yielded EHPMA (57%) as a colorless liquid.

^{1}H-NMR (500 MHz, $CDCl_3$ δ): 6.14 (m_c, 1H, trans-*H*); 5.59 (m_c, 1H, cis-*H*); 4.22 (m_c, 2H, -COOCH_2); 4.05 (m_c, 1H, -C*H*OH); 3.60-3.41 (m, 4H, -CH_2OCH_2CH_3); 2.57 (d, 1H, -CHO*H*); 1.95 (m_c, 3H, CH_2CCH_3); 1.21 (t, 3H, -CH_2CH_3) ppm.

2.2. Synthesis of BPA

4-Hydroxybenzophenone (5.0 g, 25.2 mmol, 1 eq) and NEt_3 (4.19 mL, 30.2 mmol, 1.2 eq) were dissolved in DCM (25 mL) and cooled down to 0 °C in a tinfoil protected flask. Acryloyl chloride (2.73 mL, 30.2 mmol, 1.2 eq) was dissolved in DCM (5 mL) and added dropwise to the reaction mixture over 30 min. The mixture was stirred for 16 h at rt. The precipitating solid was filtered off and washed with DCM (3 × 20 mL). The crude reaction mixture was washed with 0.1 M HCL (50 mL), saturated Na_2CO_3 (50 mL), and saturated brine (50 mL). The combined organic layers were dried over Na_2SO_4. The solvent was removed under reduced pressure, and the raw product was purified by column chromatography (DCM:EA 4/1 v:v) and dried in high vacuum. BPA (79%) was obtained as a white solid.

^{1}H-NMR (500 MHz, $CDCl_3$ δ): 7.88 (m_c, 2H, Ar-*H*); 7.81 (m_c, 2H, Ar-*H*); 7.60 (m_c, 1H, Ar-*H*); 7.49 (m_c, 2H, Ar-*H*); 7.27 (m_c, 2H, Ar-*H*); 6.65 (dd, 1H, cis-*H*); 6.35 (dd, 1H, CH_2C*H*COO); 6.07 (d, 1H, trans-*H*) ppm.

2.3. Synthesis of BPMA

4-Hydroxybenzophenone (5.64 g, 28.5 mmol, 1.1 eq) and NEt_3 (3.9 mL, 27 mmol, 1 eq) was dissolved in DCM (25 mL) and cooled down to 0 °C in a tinfoil protected flask. Methacryloyl chloride (4.2 mL, 43 mmol, 1.6 eq) was dissolved in DCM (5 mL) and added dropwise to the reaction mixture over 20 min. The mixture was stirred for 20 h at rt. The crude reaction mixture was washed with saturated $NaHCO_3$ (150 mL), water (2 x 100 mL), and saturated brine (100 mL). The combined organic layers were dried over Na_2SO_4. The solvent was removed under reduced

pressure and the raw product was purified by double recrystallization from pentane/hexane (6/4 v:v). BPMA (46%) was obtained as a white solid.

^{1}H-NMR (400 MHz, $CDCl_3$ δ): 7.90-7.85 (m, 2H, Ar-*H*); 7.82-7.78 (m, 2H, Ar-*H*); 7.60 (m_c, 1H, Ar-*H*); 7.49 (m_c, 2H, Ar-*H*); 7.26 (m_c, 2H, Ar-*H*); 6.39 (m_c, 1H, cis-*H*); 5.81 (m_c, 1H, trans-*H*); 2.09 (m_c, 3H, CH_2CCH_3) ppm.

3. Polymer synthesis

The synthesis of **PGE-*b*-OBP**, **PGE-*b*-ACBP** and **pEHPA** was reported in previous works.[4, 6] The general synthesis route of **pEHPMA**, **pEHPMA-*b*-BP** and **pEHPA-*b*-BP** is presented in Scheme S2. Peak assignments were based on previous work.

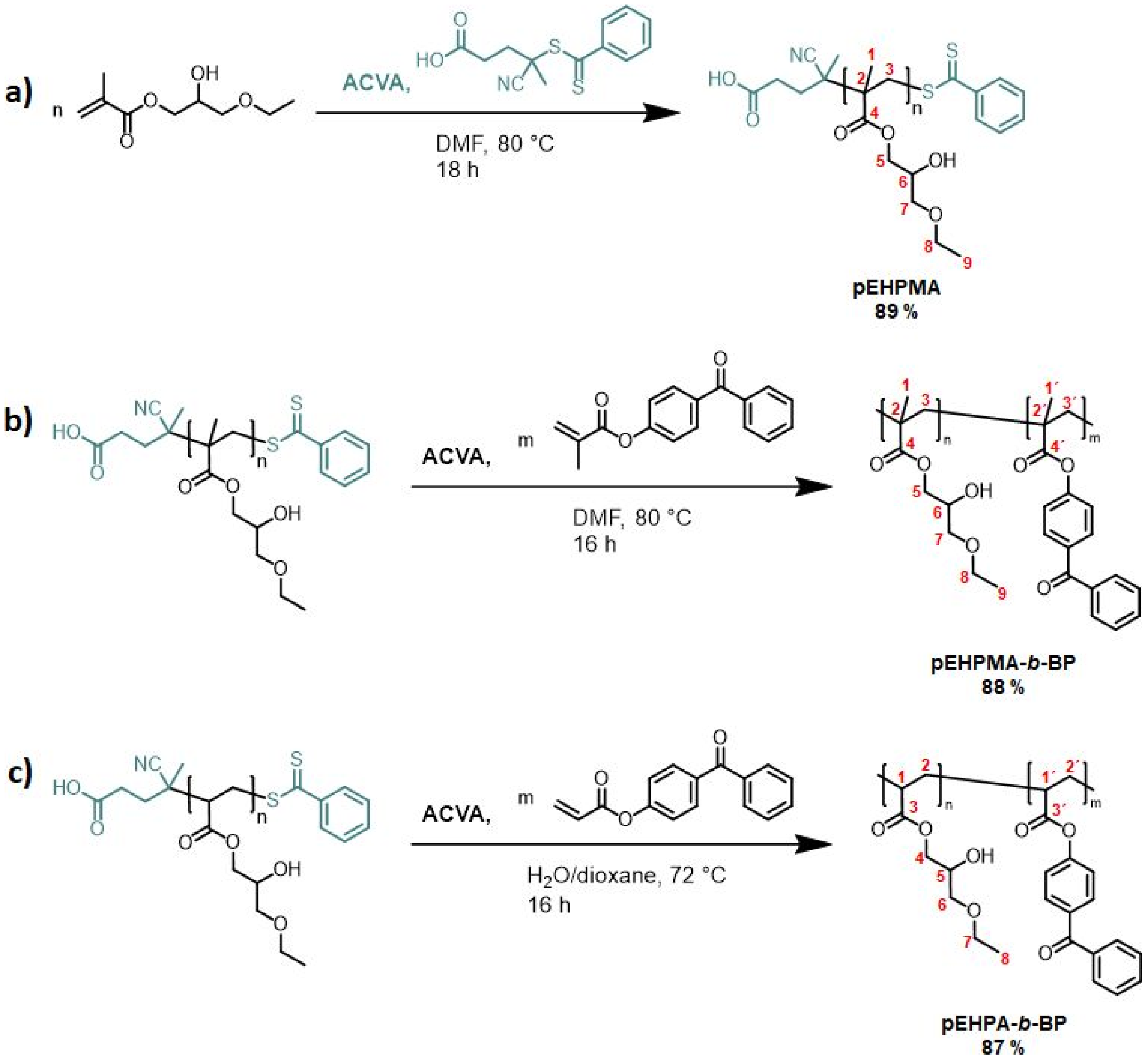

Scheme S2. Polymerization routes for **pEHPMA** (a), **pEHPMA-*b*-BP** (b), and **pEHPA-*b*-BP** (c) polymers.

3.1. Synthesis of pEHPMA

EHPMA (3.22 g, 17.1 mmol, 160 eq), radical initiator ACVA (4.3 mg, 0.015 mmol, 0.14 eq) and the RAFT agent (29.7 mg, 0.11 mmol, 1.0 eq) were dissolved in DMF (6.9 mL) at 0 °C. The solution was degassed for 20 min with argon and the reaction mixture was stirred overnight in a preheated oil bath at 80 °C. The polymerization was stopped by air exposure at 0 °C. The polymer

was precipitated from diethyl ether and dried overnight under high vacuum. pEHPMA (89 %) was obtained as a pink solid, which was used directly for the preparation of pEHPMA-*b*-BP. A small fraction was further dialyzed against methanol for the polymer analysis (NMR, GPC, turbidimetry).

^{1}H-NMR (600 MHz, MeOD, δ): 7.91, 7.59, 7.43 (RAFT Ar end-group); 4.23-3.78 (m, 3H, 4-H_2, 5-*H*); 3.76-3.41 (m, 4H, 7-H_2, 8-H_2); 2.46 (ACVA group); 2.36-1.44 (m, 2H, 3-H_2); 1.43-0.73 (m, 6H, 1-H_3, 9-H_3) ppm.

^{13}C-NMR (151 MHz; MeOD δ): 179.70- 178.10, 178.29 (4-*C*), 73.1-72.50 (7-*C*) 69.40-68.98 (5-*C*), 67.93 (8-*C*), 67.61 (6-*C*), 55.99-54.84 (3-*C*), 46.40, 46.05 (2-*C*), 19.77, 17.74 (1-*C*), 15.64 (9-*C*) ppm.

Molecular weights determined by NMR: M_n = 23.2 kDa. GPC(THF): M_n = 21700 Da, $Đ$ = 1.16.

The best results for block copolymers can be obtained, as long as the majority of the transfer agents at the end of the chains is still active. Therefore, the block copolymerization of **pEHPA** with **BPA** was proceeded directly after the polymerization in one-pot sequential manner, as described below. However, since **pEHPMA** is a solid polymer (contrary to the viscous **pEHPA**) and therefore can be prepurified via precipitation, this step was performed, before adding the benzophenone methacrylate block.

For the determination of equivalents for the block copolymerization of **pEHPMA-*b*-BP** the yield percentage of **pEHPMA** was used to determine the reacted monomer amount in the polymer for the equivalent calculation: 160 × 0.89 = 142 eq. The theoretical amount of substance was calculated by dividing the used polymer mass (1.80 g) through the molecular weight of the used **EHPMA** monomer (188.2 g/mol)

3.2. Synthesis of pEHPMA-*b*-BP

pEHPMA (1.80 g, 9.6 mmol, 142 eq), and BPMA (126.0 mg, 0.47 mmol, 7 eq) were dissolved in DMF (3.84 ml) and cooled down to 0 °C. ACVA (3.0 mg, 0.011 mmol, 0.16 eq) was added and the mixture was degassed for 25 min with argon and then stirred in a preheated oil bath overnight at 80 °C. The polymerization was stopped by air exposure at 0 °C. The raw polymer was dialyzed against methanol and dried under high vacuum. pEHPMA-*b*-BP (88%) was obtained as a pink solid.

^{1}H-NMR (700 MHz, MeOD, δ): 8.18-6.75 (Ar-*H*, BP); 4.28-3.80 (m, 3H, 4-H_2, 5-*H*); 3.78-3.38 (m, 4H, 7-H_2, 8-H_2); 2.46 (ACVA group); 2.26-1.45 (m, 2H, 3-H_2, 3´-H_2); 1.42-0.69 (m, 6H, 1-H_3, 9-H_3, 1´-H_3) ppm.

^{13}C-NMR (176 MHz; MeOD δ): 179.76- 177.49 (4-*C*, 4´-*C*), 138.55, 136.43, 133.90, 132.71, 130.96, 129.63, 122.71 (Ar-*C*, BP) 73.1-72.50 (7-*C*) 69.50-68.90 (5-*C*), 67.92 (8-*C*), 67.61 (6-*C*), 56.19-52.75 (3-*C*, 3´-*C*), 47.00 46.41, 46.06 (2-*C*, 2´-*C*), 22.09, 19.72, 17.83 (1-*C*, 1´-*C*), 15.64 (9-*C*) ppm.

Molecular weights determined by NMR: M_n = 22.6 kDa. GPC (THF): M_n = 22100 Da, $Đ$ = 1.18.

Since **pEHPA** polymers are obtained in a form of a viscous wax, rather than a solid, a purification via precipitation was not feasible. Therefore, the synthesis of **pEHPA-*b*-BP** was performed as a sequenced one-pot polymerization after verifying an appropriate conversion (95%). Amounts of the radical starter and BPA were calculated based on the remaining mixture volume and initial monomer concentration in the reaction mixture.

3.3. Synthesis of pEHPA-*b*-BP

EHPA (3.00 g, 17.22 mmol, 172 eq), radical initiator ACVA (5.6 mg, 0.2 mmol, 0.2 eq) and the RAFT agent (27.8 mg, 0.10 mmol, 1.0 eq) were dissolved in 1,4-dioxane/water mixture (10.1 mL, 1/1 v:v) at 0 °C. The mixture was degassed for 30 min with argon and then stirred in a preheated oil bath overnight at 72 °C. The reaction was paused through cooling the reaction down to 0 °C under argon atmosphere. Reaction sample (2.7 mL, equivalent to ~800 mg polymer) for NMR and GPC analysis was withdrawn. BPA (0.11 g, 0.44 mmol, 5.9 eq) and ACVA (4.1 mg, 0.015 mmol, 0.2 eq) were dissolved in 1,4-dioxane/water mixture (0.46 ml, 1/1 v:v) and added to the polymer mixture. The mixture was degassed for 30 min with argon at 0 °C and then stirred in a preheated oil bath overnight at 72 °C. The polymerization was stopped by air exposure at 0 °C. The raw polymers were dialyzed against methanol and dried under high vacuum. pEHPA (69%) and pEHPA-*b*-BP (88%) were obtained as a red wax.

pEHPA:

^{1}H-NMR (500 MHz, MeOD, δ): 8.01, 7.63, 7.46 (RAFT end-group); 4.31-4.01 (m, 2H, 4-H_2,); 4.01-3.90 (br. s, 1H, 5-*H*); 3.56 (q, 2H, 7-H_2); 3.52-3.40 (m, 2H, 6-H_2); 2.69 (ACVA group); 2.62-2.22 (m, 1H, 1-*H*); 2.22-1.42 (m, 2H, 2-H_2); 1.21 (t, 3H, 8-H_3) ppm.

Molecular weights determined by NMR: M_n = 24.6 kDa. GPC (THF): M_n = 22600 Da, $Đ$ = 1.15.

pEHPA-*b*-BP:

^{1}H-NMR (700 MHz, MeOD, δ): 8.07-7.13 (Ar-*H*, BP); 4.28-4.01 (m, 2H, 4-H_2,); 4.01-3.90 (br. s, 1H, 5-*H*); 3.56 (q, 2H, 7-H_2); 3.52-3.42 (m, 2H, 6-H_2); 2.69 (ACVA group); 2.64-2.27 (m, 1H, 1-*H*, 1´-*H*); 2.25-1.41 (m, 2H, 2-H_2, 2´-H_2); 1.21 (t, 3H, 8-H_3) ppm.

^{13}C-NMR (176 MHz; MeOD δ): 176.48- 175.92 (3-*C*, 3′-*C*), 138.72, 136.50, 133.85, 132.73, 130.97, 129.60, 128.03, 123.15 (Ar-*C*, RAFT end-group, BP), 72.80 (6-*C*), 69.51, 69.46 (5-*C*), 67.90 (7-*C*), 67.34 (4-*C*), 43.15-42.61 (1-*C*, 1′-*C*), 37.31-35.18 (2-*C*, 2′-*C*), 15.60 (8-*C*) ppm.

Molecular weights determined by NMR: M_n = 26.9 kDa. GPC (THF): M_n = 24100 Da, $Đ$ = 1.25.

3.4. Calculation of the benzophenone repeating units from the ^{1}H NMR spectra

The calculation of the benzophenone (meth)acrylate repeating units in the respective **pEHPA-*b*-BP** and **pEHPMA-*b*-BP** polymers was performed as follows: in the corresponding ^{1}H NMR spectra the methylene group located directly to the carboxyl group in the ACVA-based structure unit at 2.69 (**pEHPA**) and 2.46 (**pEHPMA**) was integrated and set to two. The integral value of the aromatic region on polymers without BP block (representing the aromatic RAFT moiety) was then subtracted from the value of the benzophenone integral value. The difference was then divided through the number of aromatic protons in the monomer (9) to yield the corresponding average BP units. The consistent presence of the BP block in the monomer was verified through UV-detected elugrams of polymers without and with BP block, as shown in Figure S1. While the RI signal stayed in the same detection range, the UV signal increased ~10-fold over the whole polymer detection range.

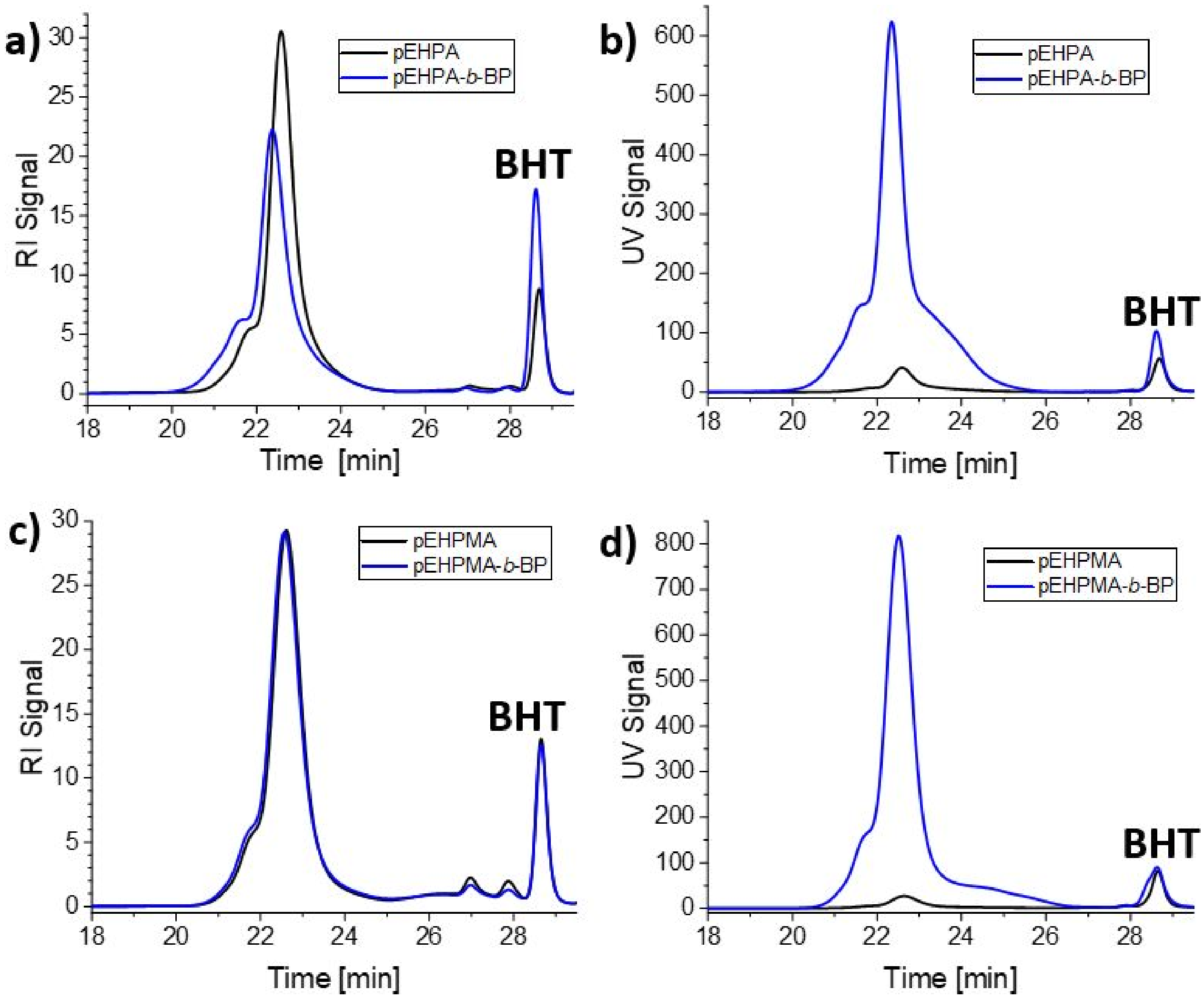

Figure S1. Overview of GPC traces of **pEHPA** and **pEHPA-*b*-BP** detected via RI (a) and UV (b) and **pEHPMA** and **pEHPMA-*b*-BP** detected via RI (c) and UV (d). The measurements were performed at room temperature with THF as eluent and butylated hydroxytoluene (**BHT**) as reference.

4. Sample preparation and analysis of the thermoresponsive behavior

4.1. Turbidimetry

The polymer samples were dissolved in water and lyophilized to remove traces of methanol. A 20 mg mL^{-1} stock solution in the respective solvent (water or PBS) was prepared and 2 mL dilutions with 10 and 5 mg mL^{-1} were prepared for analysis. Temperature-dependent turbidity curves were

measured as described in the experimental section of the main article by detecting the transmittance at 600 nm. The normalized turbidimetry curves of the **pEHPMA** homopolymer in water and PBS are presented in Figure S2. Analogous turbidimetry curves of **pEHPA-*b*-BP** and **pEHPMA-*b*-BP** block copolymers in water are presented in Figure S3.

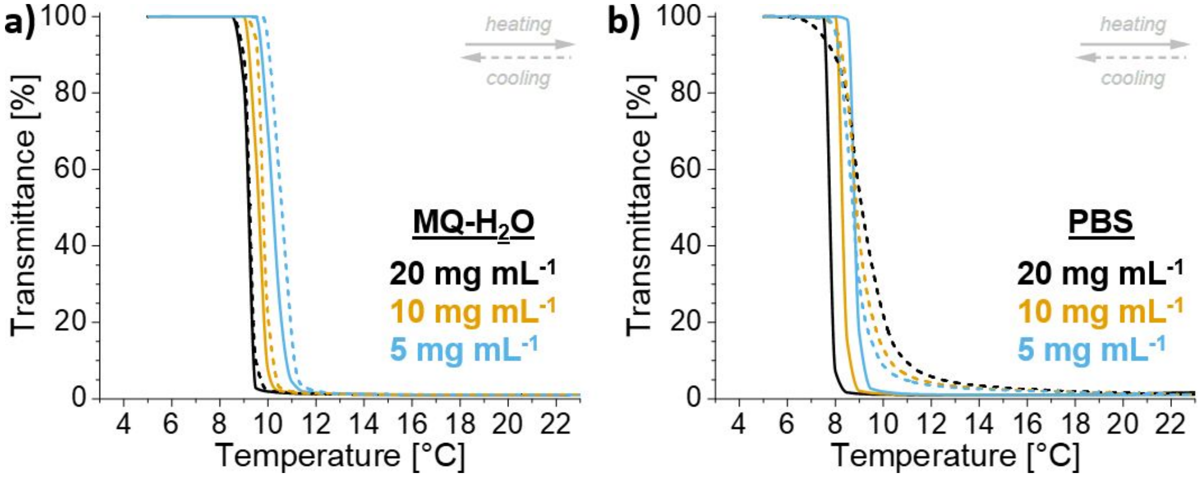

Figure S2. Representative concentration-dependent turbidimetry curves of **pEHPMA** in MQ-H_2O (a) and PBS (b) after normalization in heating and cooling cycles (n = 2 per cycle).

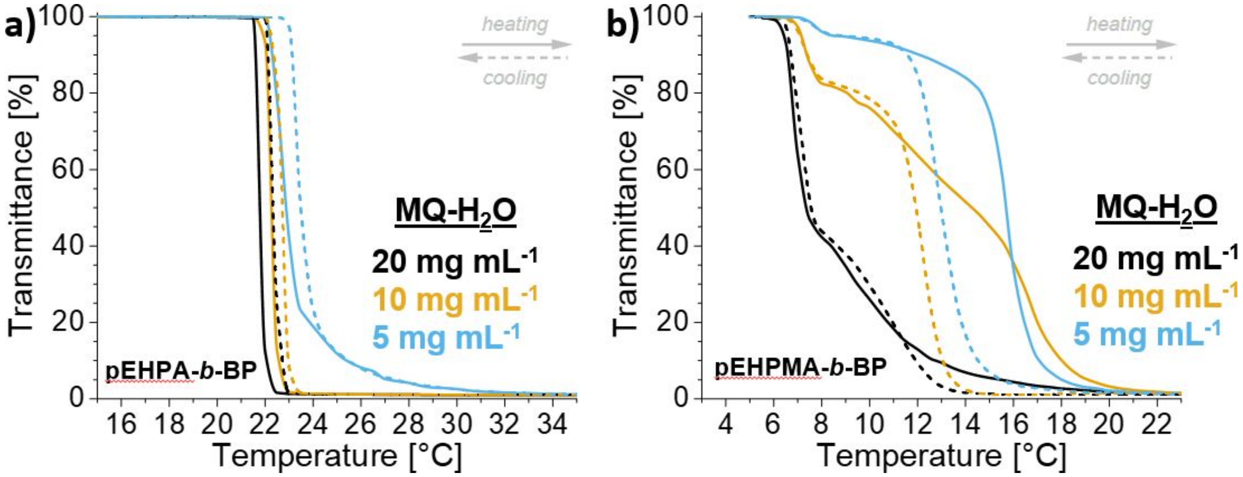

Figure S3. Representative concentration-dependent turbidimetry curves of **pEHPA-*b*-BP** (a) and **pEHPMA-*b*-BP** (b) in MQ-H_2O after normalization in heating and cooling cycles (n = 2 per cycle).

4.2. DLS measurements and intensity distribution of polymer samples

The prepared samples for turbidimetry measurements were additionally investigated by DLS. The obtained size distribution curves by intensity in aqueous solutions are shown in Figure S4 for **pEHPMA** and in Figure S5 for **pEHPA-*b*-BP** and **pEHPMA-*b*-BP**.

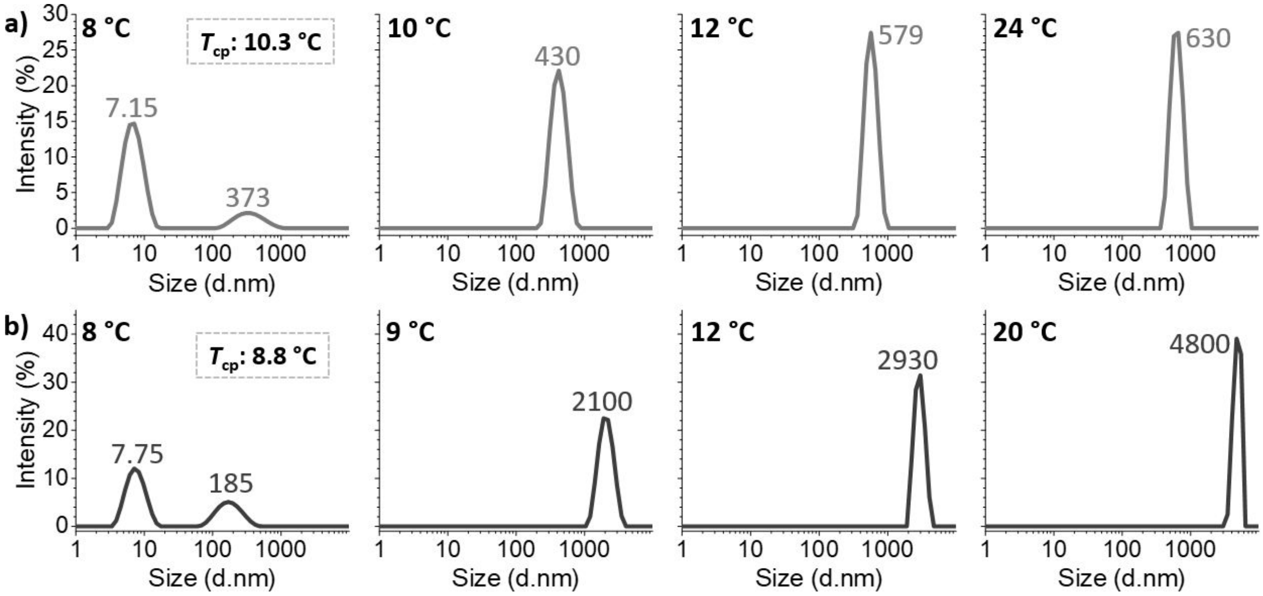

Figure S4. Temperature-dependent size distributions obtained from DLS measurements of **pEHPMA** in MilliQ-H_2O (a) and PBS (b).

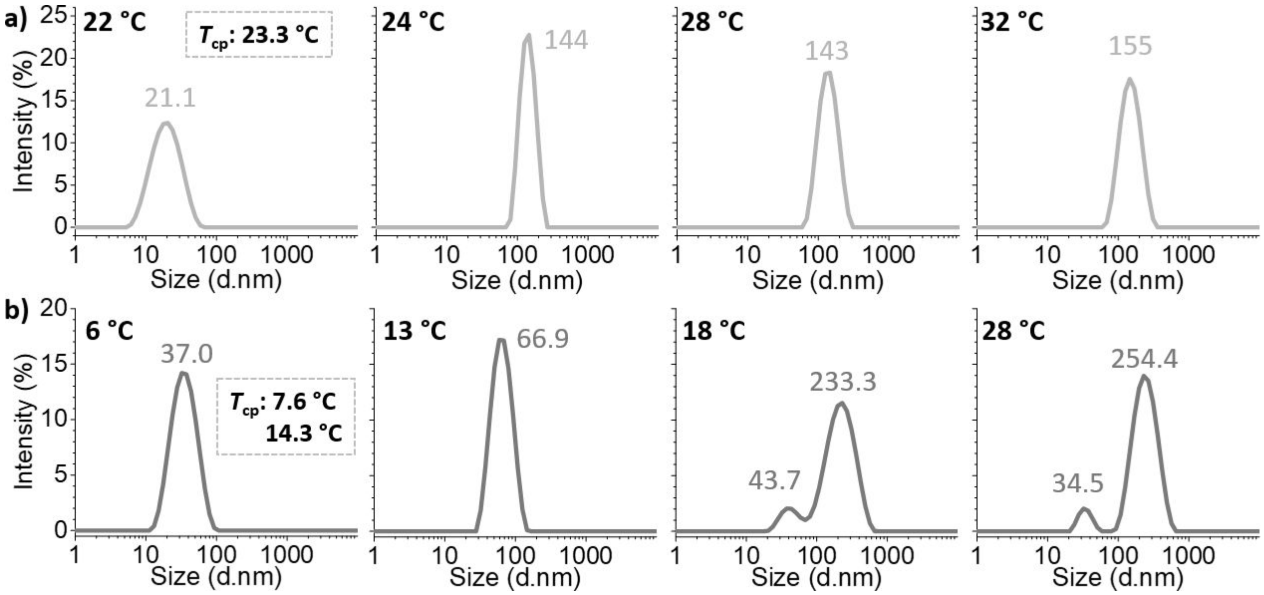

Figure S5. Temperature-dependent size distributions obtained from DLS measurements of **pEHPA-*b*-BP** (a) and **pEHPMA-*b*-BP** (b) in MilliQ-H_2O.

4.3. Temperature-dependent pEHPMA ^{1}H NMR measurements

The NMR spectra were acquired as described in the experimental section of the main manuscript. The ^{1}H spectra of **pEHPMA** in D_2O in the temperature range of 8-25 °C are shown in Figure S6.

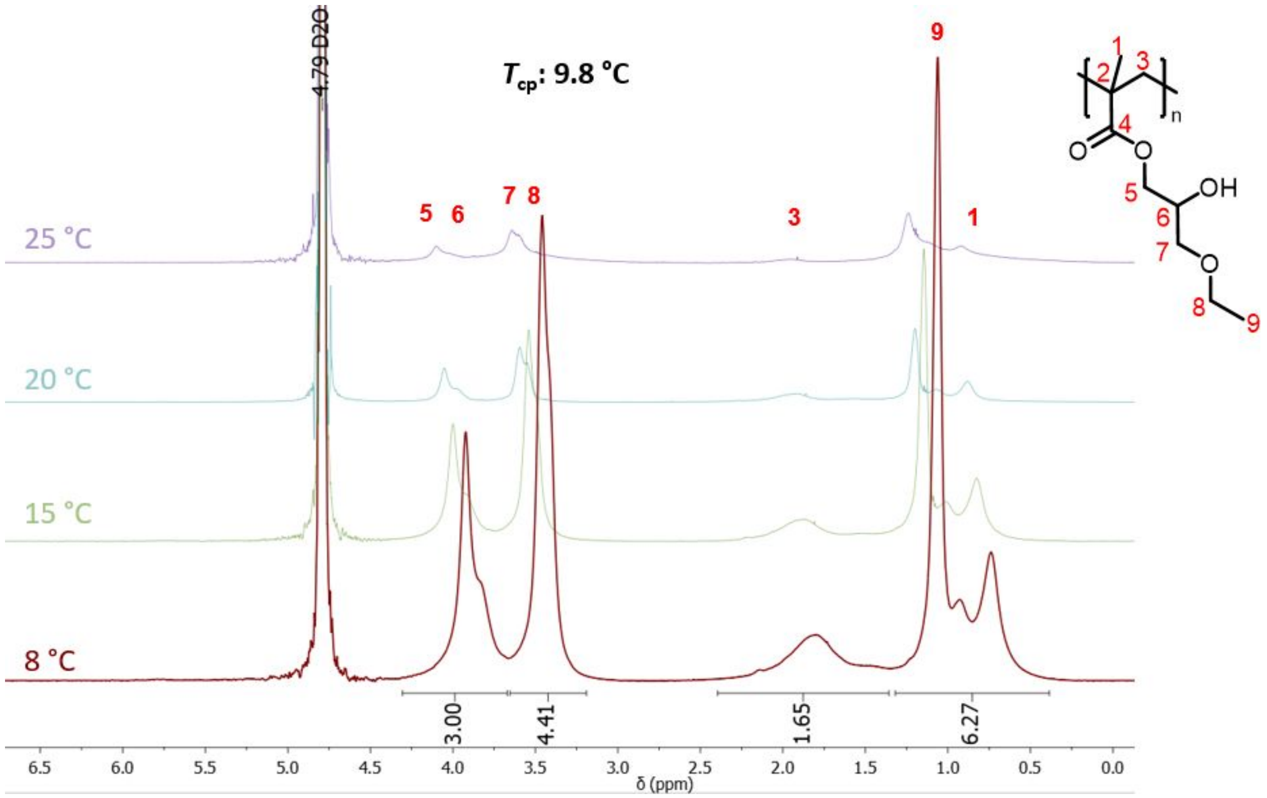

Figure S6. Temperature-dependent ^{1}H NMR spectra (D_2O, 600 MHz) of **pEHPMA** at a concentration of 10 mg mL^{-1} (T_{cp}: 9.8 °C). The spectra were referenced to the solvent peak of D_2O at 8 °C at 4.79 ppm. Peaks were assigned with numbers according to the illustrated chemical structure.

The ^{1}H NMR spectra of **pEHPMA** solution in Figure S6 show a slight temperature-dependent shift of the chemical shifts of the polymer signals. More importantly, a uniform transition can be observed through the gradual broadening of the proton signals from the methacrylic backbone as well as the γ-ethoxy-β-hydroxy-side chain. This hints towards a dehydration and aggregation of the polymer chains, which causes a significant mobility loss, resulting in the increased signal broadening along with declining signal intensity.[7, 8]

5. Comparison of homo- and block copolymer adsorption on PS

To estimate the directed adsorption potential of **pEHPA-*b*-BP** and **pEHPMA-*b*-BP**, comparative dry layer thickness measurements were performed after block- and homopolymer adsorption (c = 250 µg mL^{-1}) under established conditions, and UV-irradiation (Figure S7a). Furthermore, the resulting contact angles after the extraction of the irradiated **pEHPA** and **pEHPMA** substrates were compared to pristine PS surfaces since no layer thickness was detected after the extraction of **pEHPA** and **pEHPMA** surfaces (Figure S7b).

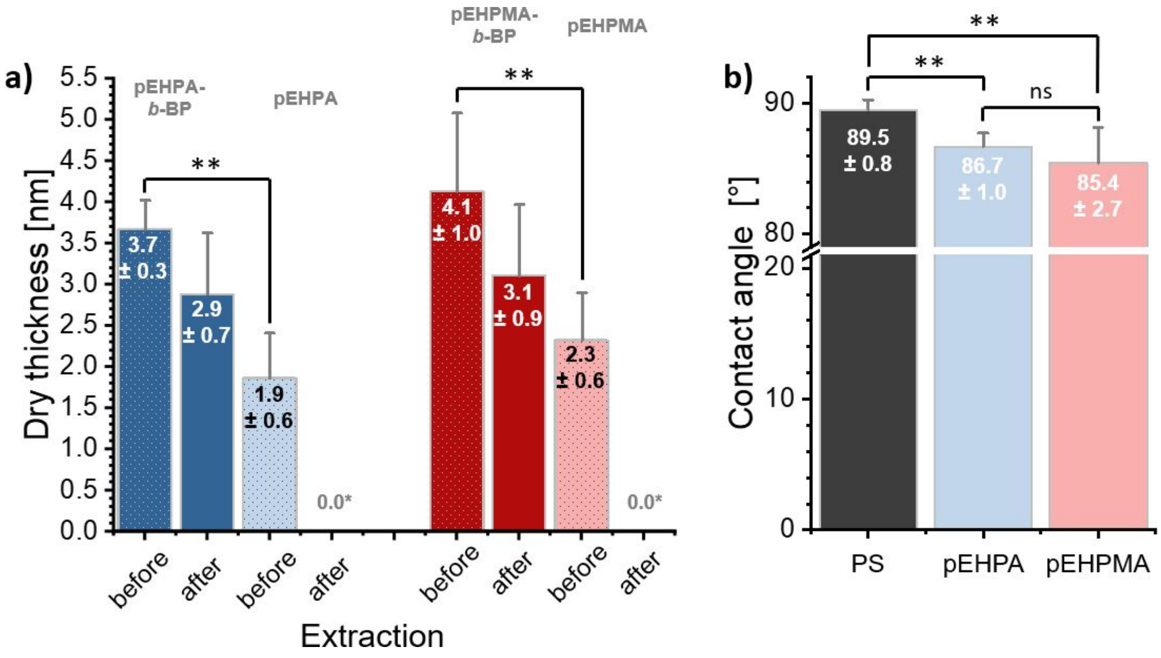

Figure S7. Comparison of surface characteristics of block copolymer and homopolymer grafted PS surfaces. (a) Comparative dry layer thickness of adsorbed (c = 250 µg mL^{-1}) and UV irradiated **pEHPA-*b*-BP** and **pEHPA** as well as **pEHPMA-*b*-BP** and **pEHPMA** coatings before and after extraction. (b) Comparative water contact angles of native PS substrates and extracted PS substrates after **pEHPA** or **pEHPMA** homopolymer adsorption ($n \geq 3$). *Measured values were below the detection limit of the spectroscopic ellipsometer.

5.1. Estimation of the chain overlap parameters based on polymer theory

In order to estimate the configuration of the surface tethered polymer chains in aqueous solution above and below their cloud point temperature T_{cp}, we calculated the theoretical degree of chain overlap. Therefore, the ratio of the Flory radius R_f of the polymer chains in a good ($R_{f,\ good}$) and bad solvent ($R_{f,\ bad}$), representing the conditions below and above the cloud point in water, and the anchor distance (l) of the chains on the surface was calculated. In previous work, we showed that the estimation of water as a good solvent is valid by comparing hydrodynamic radii of **pEHPA** in water derived from DLS data below T_{cp} to the theoretical Flory radii in a good solvent.[4, 9] Furthermore, we assume water as a bad solvent above the T_{cp} due to chain aggregation observed in turbidimetry and DLS measurements. Flory radii were calculated in a good ($R_{f,\ good} = N^{3/5}a$) and bad ($R_{f,\ bad} = N^{1/3}a$) solvent with N as the number of repeating units in the polymer chain and a as the segment chain length.[10] The number of repeating units N was calculated as the ratio of the polymer's molecular weight M_n to the respective monomer weight of **EHPA** (174.2 g/mol) or **EHPMA** (188.2 g/mol). The segment length was estimated to a = 0.25 nm as a typical value for vinyl-based polymers such as **pNIPAM**.[11] The respective anchor distance l was calculated using the dry thickness values (h) and approximating the density of **pEHPA** and **pEHPMA** to the density of **pNIPAM** (ρ_{pNIPAM} = 1.2 g cm^{-3}).[12, 13] Furthermore, a model with homogeneous distribution of circular chains on a square surface area was assumed, which is frequently used in surface science.[14, 15] This allows to calculate the anchor distance l with the chain grafting density (GD) from the layer thickness h, the polymer density ρ, the Avogadro number N_A, and the molecular weight M_n of the polymer as follows:

$$l = \sqrt{\frac{4}{\pi}\frac{1}{\mathrm{GD}}} \qquad \text{with grafting density (GD):} \qquad \mathrm{GD} = \frac{h \cdot \rho_{\mathrm{polymer}} \cdot \mathrm{N_A}}{M_\mathrm{n}}$$

The parameters for estimating the grafting density $2R_f\ l^{-1}$ of the surface tethered chains in the extracted polymer layers in a good and a bad solvent are shown in Table S2. The standard deviations of the chain overlap values were calculated based on the standard deviations of the layer thickness using Gaussian propagation of error.

Table S1. Polymer and coating parameters as described and defined in the text for the estimation of the degree of chain overlap of grafted polymers in a bad and a good solvent.

**Polymer** (concentration [µg mL^{-1}])	N	$R_{f,\,good}$ [nm]	$R_{f,\,bad}$ [nm]	h [nm]	GD [nm^{-3}]	l [nm]	$2R_{f,\,good}\ l^{-1}$	$2R_{f,\,bad}\ l^{-1}$
**pEHPA-*b*-BP** (250)	138.3	4.81	1.29	2.9 ± 0.7	0.086	3.84	2.51 ± 0.61	0.67 ± 0.16
**pEHPA-*b*-BP** (62.5)	138.3	4.81	1.29	1.9 ± 0.6	0.057	4.72	2.04 ± 0.64	0.55 ± 0.17
**pEHPMA-*b*-BP** (250)	117.4	4.36	1.22	3.1 ± 1.9	0.101	3.54	2.46 ± 0.71	0.69 ± 0.20
**pEHPMA-*b*-BP** (62.5)	117.4	4.36	1.22	2.1 ± 0.7	0.069	4.31	2.03 ± 0.68	0.57 ± 0.19

6. Surface topography and thermoresponsive behavior of brushes on PS surface

The topography of bare and brush-coated PS surfaces on silicon wafers were probed via AFM measurements, as described in the Materials and Methods section. Both brush coatings uniformly cover the PS surface, resulting in a reduced roughness compared to the pristine PS surface, as depicted through the correlated height profile. The corresponding images and height profiles are shown in Figure S8.

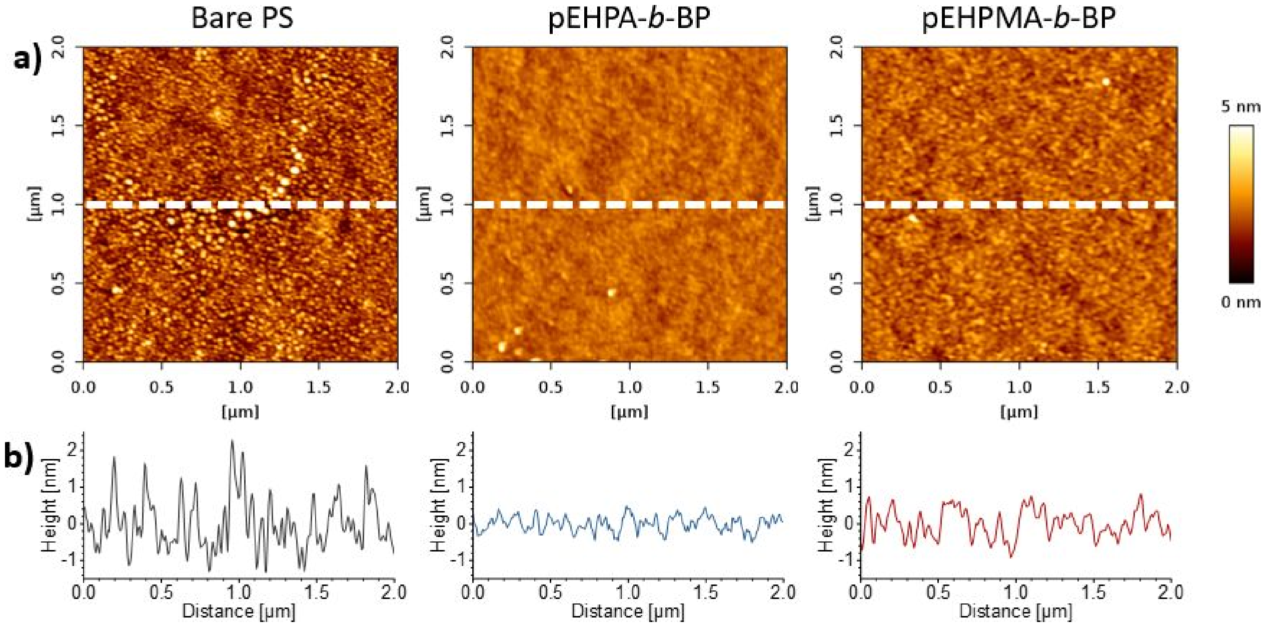

Figure S8. Representative AFM images of bare PS, **pEHPA-*b*-BP** and **pEHPMA-*b*-BP** coated PS on silicon substrates obtained in tapping mode in air (a) and the derived height profiles recorded over the dashed white lines in the AFM pictures (b).

The thermoresponsive behavior of the **pEHP[M]A-*b*-BP** brush layers was confirmed by temperature-dependent QCM-D measurements of brush-coated PS layers on silicon QCM-D sensors, similar to previous reports.[2, 16-18] The preliminary measurement of non-functionalized PS-coated QCM-D sensors under identical conditions allows the consideration of the changes in water density and viscosity as well as temperature-dependent frequency (Δf) changes of respective QCM-D sensors. Subtracting the measured Δf values of PS-coated sensors from the respective brush-functionalized sensors (Δf differentials at each temperature point, $\Delta\Delta f$) allowed us to deduce the phase transition of the brush system.[2, 19] Representative time-dependent measurements of bare PS and brush-functionalized surfaces while quasi-statically ramping the temperature and the respective temperature-dependent $\Delta\Delta f$ values are shown in Figure S9.

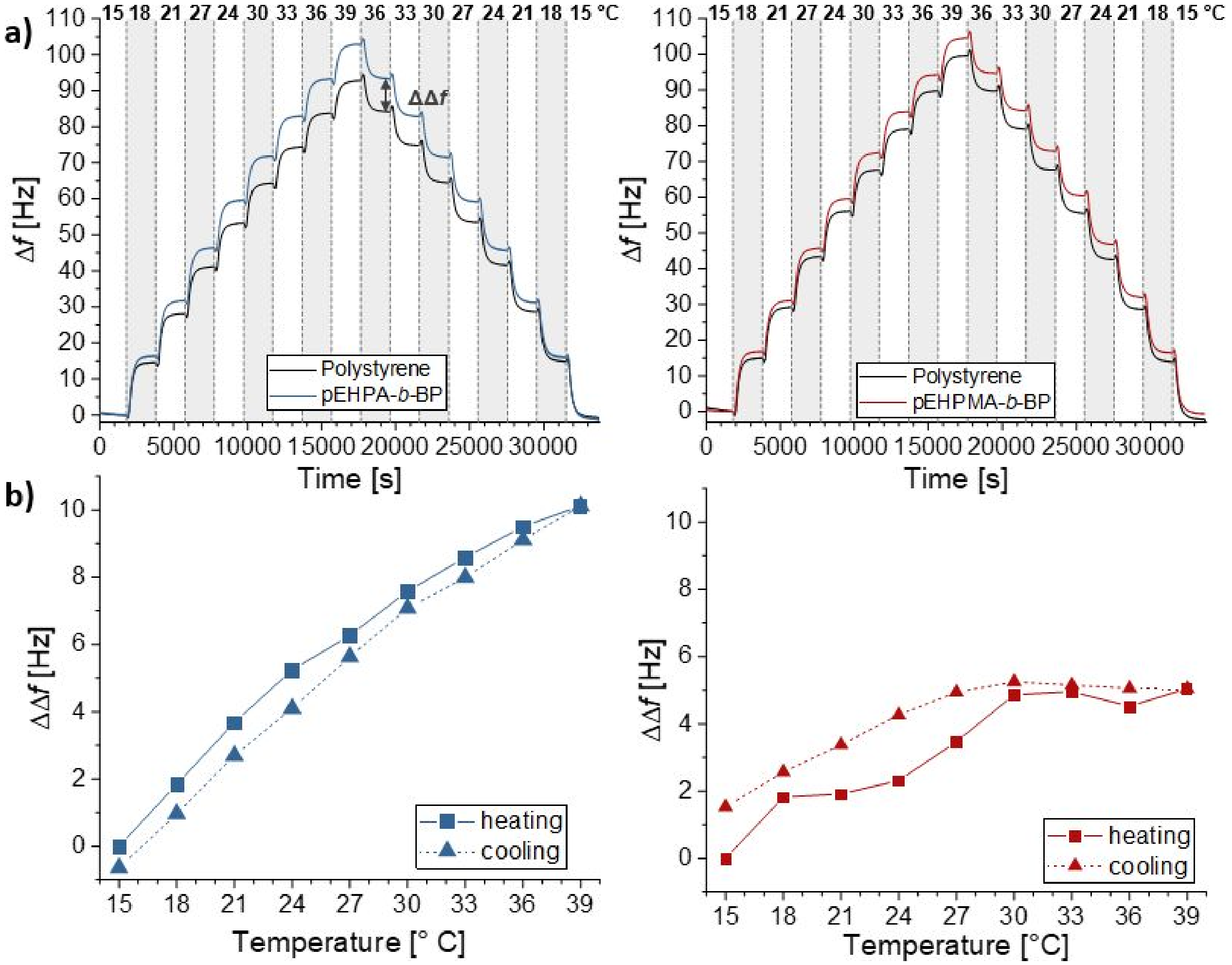

Figure S9. (a) Representative images of quasi-static temperature ramp measurements of bare and **pEHP[M]A-*b*-BP** coated PS surfaces on silicon QCM-D sensors including a visual depiction of the $\Delta\Delta f$ differential range in the **pEHPA-*b*-BP** diagram. (b) Corresponding $\Delta\Delta f$ values between the **pEHP[M]A-*b*-BP** coated and bare PS sensors, showing the (de)hydration behavior of the corresponding brush. Derived values connected to guide the eye (n = 2).

pEHPA-*b*-BP brush coatings dehydrate uniformly and reversibly in the probed temperature range. Notably, this behavior aligns well with the microscopic dehydration of polymer chains in solution, which was determined to proceed up to 40 °C *via* ^{13}C NMR spectroscopy.[4] Due to the continuous transition, a discrete phase transition temperature cannot be deduced. A pronounced phase

transition, however, is clearly observable in the probed range, thus enabling potential cell sheet fabrication.

The transition of **pEHPMA-*b*-BP** brush coatings levels at ~30 °C, since the $\Delta\Delta f$ values remain constant at higher temperatures, indicating no brush-dependent changes in hydration above 30 °C. The phase transition on the surface extends beyond the macroscopically determined sharp T_{cp} values (6-14 °C, **Table 2**) in solution, in agreement with the microscopic dehydration of polymer chains in solution up to 25 °C, as shown *via* temperature-dependent ^{13}C NMR spectroscopy (**Figure 3** in the manuscript). Upon cooling, brush rehydration seems to proceed slower, resulting in a small hysteresis between heating and cooling cycles. Hampered water intake could be caused by hydrophobic interactions of the collapsed **pEHPMA-*b*-BP** brush and the underlying PS substrate.[20] However, this observation needs to be interpreted with caution, due to the generally small $\Delta\Delta f$ differences between PS and **pEHPMA-*b*-BP** functionalized surfaces.

7. Additional cell adhesion images

The adhesion and proliferation of human dermal fibroblasts seeded on polymer brush coated substrates and TCPS controls at a density of 43 x 10^5 cells cm^{-2} was observed up to 72 h after seeding. Representative phase contrast images after 48 and 72 h are shown in Figure S8.

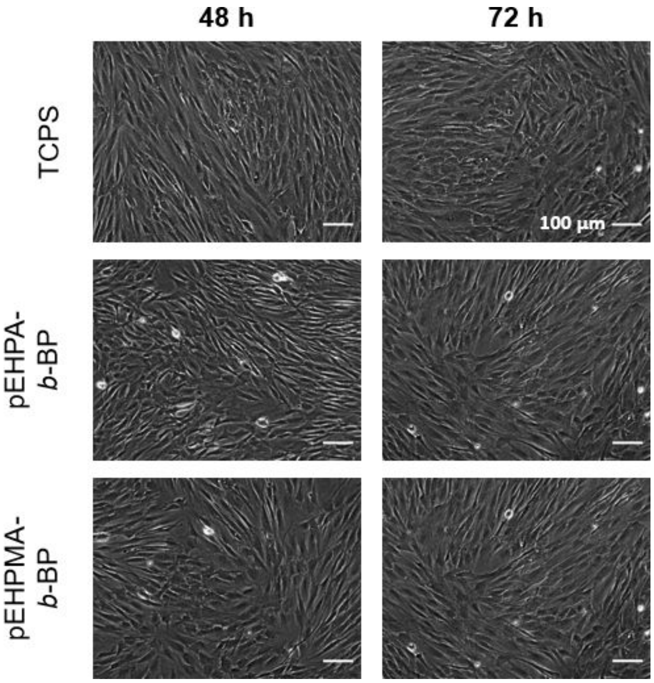

Figure S10. Representative phase contrast images of human dermal fibroblasts on **TCPS** (top), **pEHPA-*b*-BP** (middle), and **pEHPMA-*b*-BP** (bottom) coated PS substrates 48 and 72 h after seeding at a density of 43×10^5 cells cm^{-2}.

8. Surface parameters of brush-coated QCM-D chips

The average dry layer thickness of **pEHPA-*b*-BP** and **pEHPMA-*b*-BP** coatings on PS-coated QCM-D chips, used to quantify the areal mass of adsorbed proteins are shown in Figure S9a. Corresponding water contact angles on these brush coatings, along with the values of the used **TCPS** reference, are shown in Figure S9b. Representative real-time frequency curves of protein adsorption measurements conducted *via* QCM-D are shown in Figure S10.

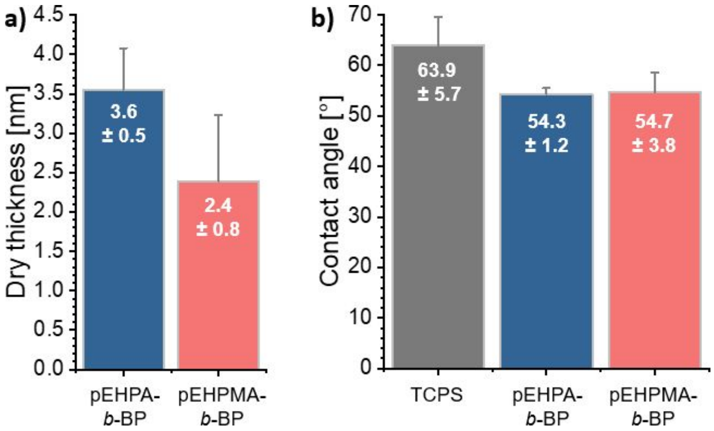

Figure S11. Dry layer thickness of **pEHPA-*b*-BP** and **pEHPMA-*b*-BP** brush coatings on PS-coated QCM-D chips (a) and their corresponding water contact angles, along with the values for TCPS controls (b) ($n \geq 3$).

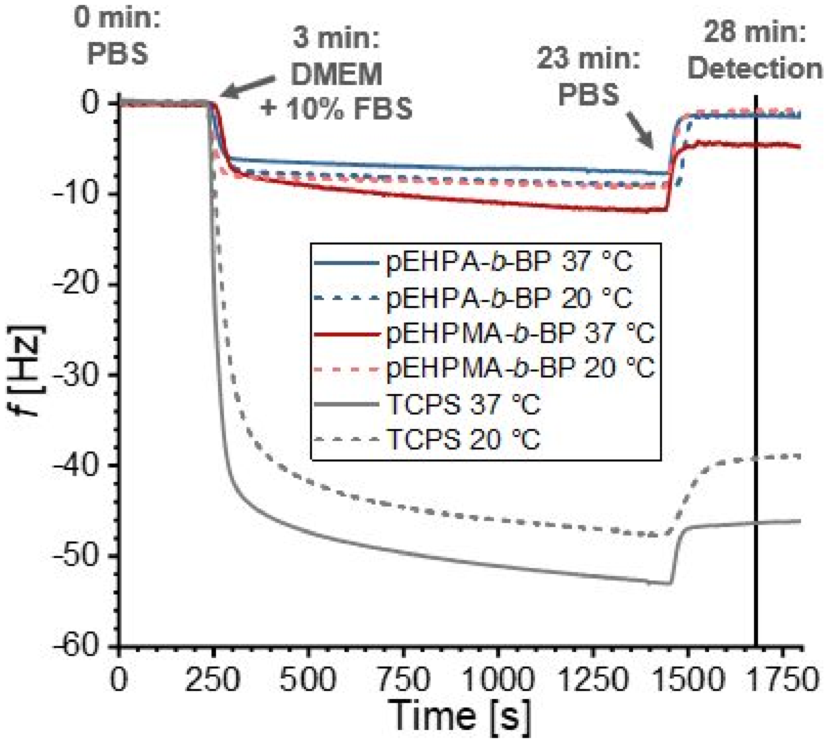

Figure S12. Representative frequency curves of the protein adsorption from DMEM cell culture medium supplemented with 10% FCS obtained from QCM-D measurements at 37 and 20 °C on brush coatings and TCPS controls.

9. Viability of thermally detached cells

To assess the viability of thermally detached confluent sheets from **pEHPA-*b*-BP** surfaces, the cells were trypsinized after detachment parallel to confluent cells cultured on TCPS substrates and harvested conventionally by trypsinization. Dead cells were stained with propidium iodide, and all cells were counted via flow cytometry. The counted values of all measurements on **pEHPA-*b*-BP** and TCPS (n = 3, respectively), along with the threshold for the dead cells, are shown in Figure S11. The average percentage of dead cells and standard deviation are embedded.

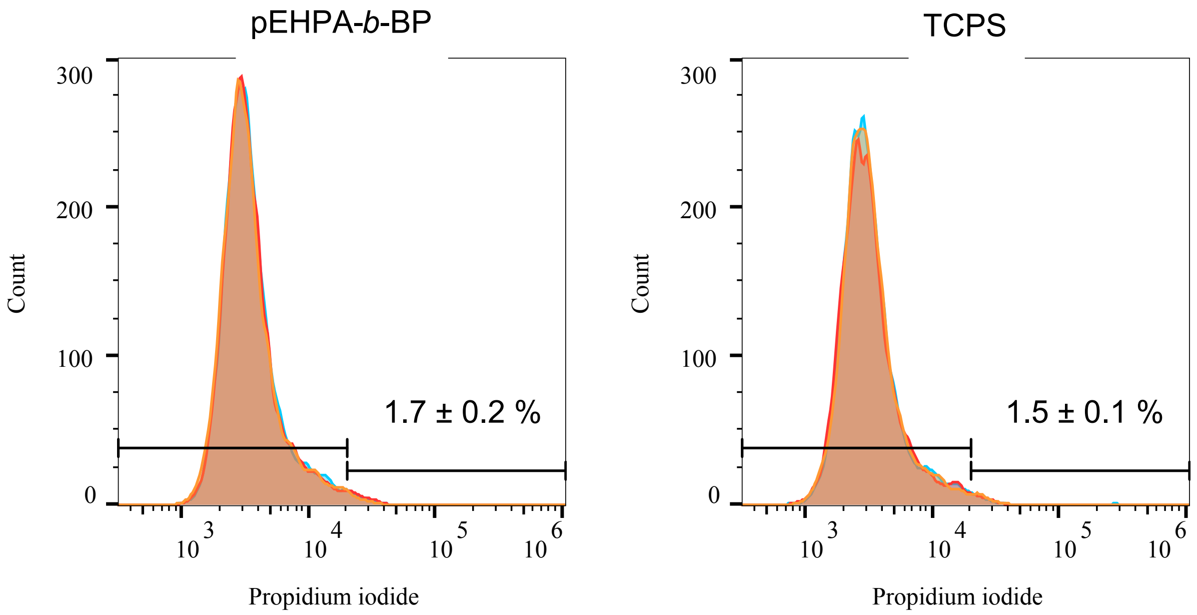

Figure S13. Flow cytometry counts of cells (red, orange, blue) thermally detached from **pEHPA-*b*-BP** (left) and trypsinized from TCPS (right). Number values indicate the mean percentage of dead cells along with the standard deviation (n = 3).

10. Storage stability of coated substrates

In order to distinguish between the impact of the sterilization treatment and the storage time between measurements of pristine and sterilized surfaces, pristine brush surfaces were stored under ambient conditions until the sterilized samples were returned and analyzed (14-18 d). The non-sterilized samples were then treated identically (extraction in ethanol for 16 h) for comparison. The thickness and contact angle differences of **pEHPA-*b*-BP**, **PGE-*b*-OBP**, and **PGE-*b*-ACBP** coatings are summarized in Figure S12.

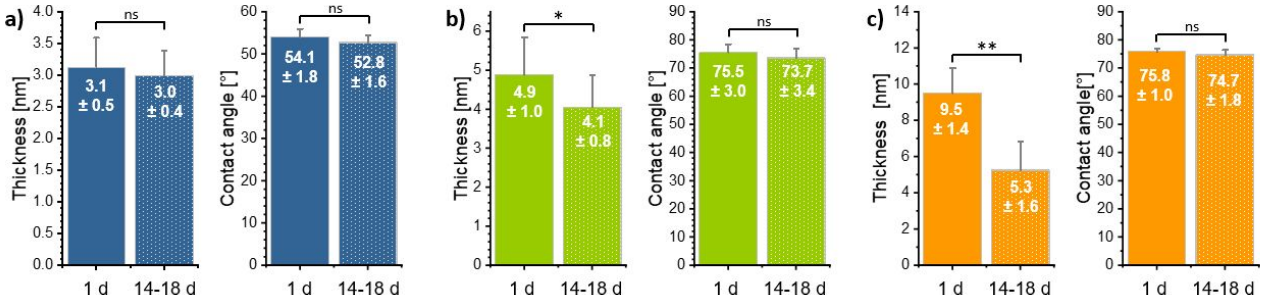

Figure S14. Thickness and contact angle values of **pEHPA-*b*-BP** (a), **PGE-*b*-OBP** (b), and **PGE-*b*-ACBP** (c) after coating preparation and extraction (1 d) and after 14-18 d of ambient storage and subsequent extraction (n ≥ 3).

11. Impact of gamma and FO treatment on CAs of pristine PS substrates

In order to access the impact of the used sterilization methods on pristine **PS** substrates, untreated **PS** was sterilized under identical conditions to the brush coatings and extracted in ethanol for 16 h after sterilization. The resulting CAs are shown in Figure S13.

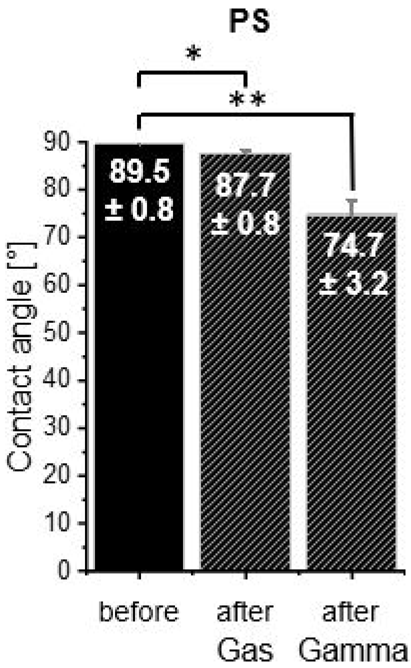

Figure S15. Contact angles of untreated and sterilized PS after ethanol extraction.

12. References

(1) Zhang, G. Study on Conformation Change of Thermally Sensitive Linear Grafted Poly(N-isopropylacrylamide) Chains by Quartz Crystal Microbalance. *Macromolecules* **2004**, *37* (17), 6553-6557. DOI: 10.1021/ma035937+.

(2) Liu, G.; Zhang, G. Collapse and swelling of thermally sensitive poly(N-isopropylacrylamide) brushes monitored with a quartz crystal microbalance. *J Phys Chem B* **2005**, *109* (2), 743-747. DOI: 10.1021/jp046903m.

(3) Stöbener, D. D.; Uckert, M.; Cuellar-Camacho, J. L.; Hoppensack, A.; Weinhart, M. Ultrathin Poly(glycidyl ether) Coatings on Polystyrene for Temperature-Triggered Human Dermal Fibroblast Sheet Fabrication. *ACS Biomaterials Science & Engineering* **2017**, *3* (9), 2155-2165. DOI: 10.1021/acsbiomaterials.7b00270.

(4) Schweigerdt, A.; Stöbener, D. D.; Schäfer, A.; Kara, S.; Weinhart, M. Impact of Amphiphilicity Balance in Hydroxy-Functional, Isomeric, Thermoresponsive Poly(meth)acrylates. *Macromolecules* **2023**, *56* (21), 8602-8613. DOI: 10.1021/acs.macromol.3c01251.

(5) Kecici, Z.; Babaoglu, S.; Temel, G. Methacrylated benzophone as triple functional compound for the synthesis of partially crosslinked copolymers. *Progress in Organic Coatings* **2018**, *115*, 138-142. DOI: 10.1016/j.porgcoat.2017.11.015.

(6) Stobener, D. D.; Weinhart, M. Thermoresponsive Poly(glycidyl ether) Brush Coatings on Various Tissue Culture Substrates-How Block Copolymer Design and Substrate Material Govern Self-Assembly and Phase Transition. *Polymers (Basel)* **2020**, *12* (9). DOI: 10.3390/polym12091899.

(7) Han, S.; Hagiwara, M.; Ishizone, T. Synthesis of Thermally Sensitive Water-Soluble Polymethacrylates by Living Anionic Polymerizations of Oligo(ethylene glycol) Methyl Ether Methacrylates. *Macromolecules* **2003**, *36* (22), 8312-8319. DOI: 10.1021/ma0347971.

(8) Deshmukh, M. V.; Vaidya, A. A.; Kulkarni, M. G.; Rajamohanan, P. R.; Ganapathy, S. LCST in poly(N-isopropylacrylamide) copolymers: high resolution proton NMR investigations. *Polymer* **2000**, *41* (22), 7951-7960. DOI: 10.1016/s0032-3861(00)00174-9.

(9) Kubota, K.; Fujishige, S.; Ando, I. Solution Properties of Poly(N-isopropylacrylamide) in Water. *Polym J* **1990**, *22* (1), 15-20. DOI: 10.1295/polymj.22.15.

(10) Israelachvili, J. N. *Intermolecular and Surface Forces*; Elsevier Science, 2011.

(11) Ohta, H.; Ando, I.; Fujishige, S.; Kubota, K. A 13C PST/MAS NMR study of poly (N-isopropylacrylamide) in solution and in the gel phase. *Journal of Molecular Structure* **1991**, *245* (3-4), 391-397. DOI: 10.1016/0022-2860(91)87113-v.

(12) Lele, A. K.; Hirve, M. M.; Badiger, M. V.; Mashelkar, R. A. Predictions of Bound Water Content in Poly(N-isopropylacrylamide) Gel†. *Macromolecules* **1997**, *30* (1), 157-159. DOI: 10.1021/ma950894l.

(13) Ishida, N.; Biggs, S. Direct observation of the phase transition for a poly(N-isopropylacryamide) layer grafted onto a solid surface by AFM and QCM-D. *Langmuir* **2007**, *23* (22), 11083-11088. DOI: 10.1021/la701461b.

(14) Plunkett, K. N.; Zhu, X.; Moore, J. S.; Leckband, D. E. PNIPAM chain collapse depends on the molecular weight and grafting density. *Langmuir* **2006**, *22* (9), 4259-4266. DOI: 10.1021/la0531502.

(15) Kim, M.; Schmitt, S.; Choi, J.; Krutty, J.; Gopalan, P. From Self-Assembled Monolayers to Coatings: Advances in the Synthesis and Nanobio Applications of Polymer Brushes. *Polymers* **2015**, *7* (7), 1346-1378. DOI: 10.3390/polym7071346.

(16) Stobener, D. D.; Weinhart, M. "Fuzzy hair" promotes cell sheet detachment from thermoresponsive brushes already above their volume phase transition temperature. *Biomater Adv* **2022**, *141*, 213101. DOI: 10.1016/j.bioadv.2022.213101 From NLM Publisher.

(17) Adam, S.; Koenig, M.; Rodenhausen, K. B.; Eichhorn, K.-J.; Oertel, U.; Schubert, M.; Stamm, M.; Uhlmann, P. Quartz crystal microbalance with coupled spectroscopic ellipsometry-study of temperature-responsive polymer brush systems. *Appl Surf Sci* **2017**, *421*, 843-851. DOI: 10.1016/j.apsusc.2017.02.078.

(18) Schweigerdt, A.; Heinen, S.; Stobener, D. D.; Weinhart, M. Grafting Density-Dependent Phase Transition Mechanism of Thermoresponsive Poly(glycidyl ether) Brushes: A Comprehensive QCM-D Study. *Langmuir* **2021**, *37* (23), 7087-7096. DOI: 10.1021/acs.langmuir.1c00695.

(19) Laloyaux, X.; Mathy, B.; Nysten, B.; Jonas, A. M. Surface and bulk collapse transitions of thermoresponsive polymer brushes. *Langmuir* **2010**, *26* (2), 838-847. DOI: 10.1021/la902285t.

(20) Stobener, D. D.; Weinhart, M. On the foundation of thermal "Switching": The culture substrate governs the phase transition mechanism of thermoresponsive brushes and their performance in cell sheet fabrication. *Acta Biomater* **2021**, *136*, 243-253. DOI: 10.1016/j.actbio.2021.09.012 From NLM Medline.
